# Modeling transcriptional regulation using gene regulatory networks based on multi-omics data sources

**DOI:** 10.1186/s12859-021-04126-3

**Published:** 2021-04-19

**Authors:** Neel Patel, William S. Bush

**Affiliations:** 1 Department of Nutrition, Case Western Reserve University, Cleveland, OH USA; 2 Department of Population and Quantitative Health Sciences, Case Western Reserve University, Cleveland, OH USA

**Keywords:** Transcription factors, Gene expression, Machine learning, Gene regulatory network

## Abstract

**Background:**

Transcriptional regulation is complex, requiring multiple *cis* (local) and *trans* acting mechanisms working in concert to drive gene expression, with disruption of these processes linked to multiple diseases. Previous computational attempts to understand the influence of regulatory mechanisms on gene expression have used prediction models containing input features derived from *cis* regulatory factors. However, local chromatin looping and *trans-*acting mechanisms are known to also influence transcriptional regulation, and their inclusion may improve model accuracy and interpretation. In this study, we create a general model of transcription factor influence on gene expression by incorporating both *cis* and *trans* gene regulatory features.

**Results:**

We describe a computational framework to model gene expression for GM12878 and K562 cell lines. This framework weights the impact of transcription factor-based regulatory data using multi-omics gene regulatory networks to account for both *cis* and *trans* acting mechanisms, and measures of the local chromatin context. These prediction models perform significantly better compared to models containing *cis*-regulatory features alone. Models that additionally integrate long distance chromatin interactions (or chromatin looping) between distal transcription factor binding regions and gene promoters also show improved accuracy. As a demonstration of their utility, effect estimates from these models were used to weight *cis*-regulatory rare variants for sequence kernel association test analyses of gene expression.

**Conclusions:**

Our models generate refined effect estimates for the influence of individual transcription factors on gene expression, allowing characterization of their roles across the genome. This work also provides a framework for integrating multiple data types into a single model of transcriptional regulation.

**Supplementary Information:**

The online version contains supplementary material available at 10.1186/s12859-021-04126-3.

## Introduction

Dysregulation of transcription and gene expression has been linked to conditions such as diabetes [1], different subtypes of cancer [2] and neurological [3], autoimmune [4] and developmental disorders [5]. However, due to the complexity of the process of transcriptional regulation in eukaryotes, the mechanistic underpinnings of many of these diseases are yet unknown. Databases such as the Encyclopedia of DNA elements (ENCODE) [6], FANTOM5 [7] and gene expression omnibus (GEO) [8] have provided researchers with the opportunity to explore gene expression regulation using computational methods. These databases contain information about the binding sites of transcription factors (TFs), coordinates of regulatory elements such as promoters and enhancers as well as epigenetic markers, and changes in expression patterns in response to external stimuli on a genome-wide level. Furthermore, with significant advancement in sequencing technology in the past decade, more and more genetic variants associated with the aforementioned disorders have been identified [9–13]. A majority of these variants are present within the transcriptional regulatory elements and TF binding sites (TFBS) [9–13]. However, despite the availability of the epigenomic, transcriptomic, and genomic data, there is a dearth of integrative algorithms that consolidate these data types into models of regulatory impact on gene expression. Such models would provide relative weights of TF influence over gene expression, and could also be used to annotate and prioritize regulatory variants within genetic association tests for several diseases. Furthermore, knowing the relative TF weights would help in characterizing their roles in occurrence and pathogenicity of these diseases.

Current computational approaches typically model gene expression utilizing basic information corresponding to *cis*/local regulatory mechanisms such as histone modification and TF binding strengths [14–18]. Early work conducted by Ouyang et al. built linear regression models to predict gene expression in embryonic stem cells (ESCs) using TF association strengths (ChIP-Seq intensity relative to transcription start site) of 12 essential TFs and principal components to capture their “multi-collinearity” [18]. Cheng et al. [17] and Zhang et al. [15] extended this work by including ChIP-seq data for histone modifications overlapping transcription start and termination sites and applying support vector regression. Schmidt et al. [16] developed the TEPIC method to calculate TF-target gene(TG) affinity scores using a biophysical model of binding based on open chromatin assay data; using affinity scores as input features, they used regularized linear regression models to predict gene expression. More recently, deep learning models have become popular for this task [19–21], although inferring biologically relevant information from these complex models has remained a challenge. All of these approaches have produced prediction models with varying accuracy, though none of these models have attempted to incorporate additional *trans* regulatory effects such as expression levels of the TFs themselves and the co-operative interactions among TFs. Despite their important role in gene regulation, these *trans* regulatory mechanisms have largely been excluded from the modelling approaches described above due to the difficulty in quantifying their effects.

Weighted gene regulatory networks (GRNs) attempt to fill this gap by capturing information corresponding to multiple *cis* and *trans*-acting transcriptional regulatory mechanisms in the form of edge-weights between a regulator and its TG [22]. The Passing Attributes between Networks for Data Assimilation (PANDA) algorithm generates such a GRN by extracting information from heterogeneous networks built using multiple big “omics” data sources corresponding to different TF-based regulatory mechanisms [23]. Published approaches (except for a recent extension of the TEPIC framework [24]) have also not yet considered the impact of chromatin conformation on transcriptional regulation despite its increasing availability from high throughput assays such as Hi-C [25]. Condensed chromatin within the cell is heavily restructured during the process of transcription, leading to increased accessibility of gene promoters and closer physical proximity of distal transcription machinery and enhancer elements [26].

In this study, we generated a multi-omics PANDA GRN based on TF-TG features derived from multiple *cis* and *trans* acting transcriptional regulatory mechanisms to predict gene expression in the GM12878 immortalized lymphoblastoid cell line and the K562 chronic myelogenous leukemia cell line. We further derived TF feature weights in the  form of linear effect estimates from our learned models in order to characterize the individual influence of each TF on gene expression. In addition, we compared the prediction performance of models built using TF binding sites (TFBS) found within various regulatory elements such as introns, promoters and distal regulatory regions, and further assessed the impact of long distance interactions between TF binding distal regulatory elements and promoters on gene regulation by integrating Hi-C data into our GRNs and prediction models. Finally, in order to show the utility of our framework, we utilized the TF feature weights to perform rare* cis*-regulatory variants based weighted sequence kernel assocation test (SKAT) using depression genes and network (DGN) dataset for discovery [27] and the genotype tissue and expression (GTEx) dataset for replication [28]. Our in-silico prediction framework has the flexibility of including datatypes from multiple heterogeneous sources for estimating the relative influence of multiple regulatory mechanisms on gene expression. It also provides a potential blueprint for researchers of incorporating functional transcriptomic and genomic data in order to gain mechanistic understanding of diseases.

## Results

### Accounting for *trans* acting mechanisms in addition to *cis* regulatory mechanisms improved gene expression prediction significantly

**Fig. 1:**
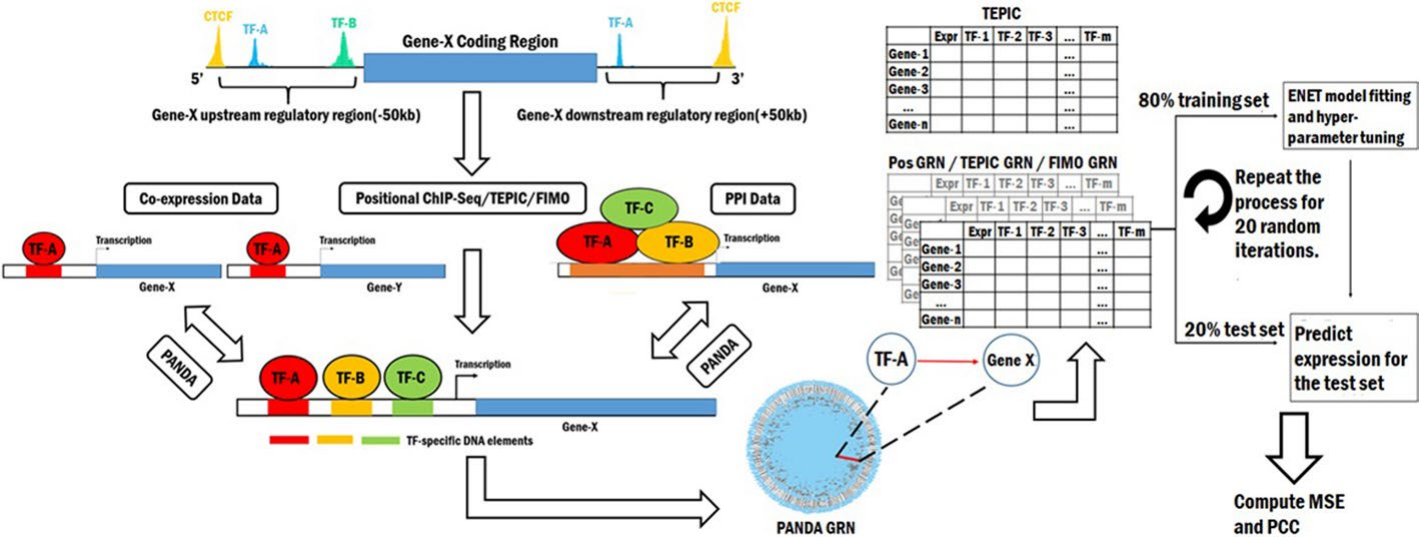
Workflow for building prediction models using multi-omics GRNs. ChIP-seq data for 153 TFs (GM12878) and 382 TFs (K562) having peaks passing the optimal irreproducible discovery rate (IDR) threshold defined by ENCODE were mapped to the regulatory region of each gene to define TFBS. The most distant CTCF peaks within a 50 Kb window upstream and downstream of the gene body were used to demarcate regulatory boundaries. Statistically significant TFBS from these regions were identified by FIMO and TEPIC based TF-TG affinity scores were calculated. PANDA GRNs were then generated using weighted and unweighted adjacency matrices. PPI data from BioGRID corresponding to TFs for each cell lines and cell line specific co-expression were obtained from GEUVADIS (GM12878) and ENCODE (K562). Elastic Net (ENET)-based regularized regression models were built from the resulting input features to predict log FPKM values (gene expression) of independent datasets for the two cell lines

We first sought to extend existing approaches for building general models of gene expression genome-wide based on TF-TG interactions. We hypothesized that accounting for *trans*-acting mechanisms in addition to *cis* acting ones would improve overall prediction of gene expression. To test this hypothesis, we first constructed GRNs using the PANDA algorithm utilizing three separate networks: a motif network, a protein–protein interaction (PPI) network and a co-expression network for GM12878 and K562 cell lines as shown in Fig. 1.

For the motif network, we first identified TFs interacting with the *cis*-regulatory region of each protein coding TG by isolating the TF ChIP-seq peaks occurring within the regulatory window demarcated by the most upstream and downstream occurring CTCF ChIP-seq peaks within a 50 Kb region surrounding the gene body (Fig. 1). We further filtered these positional TFBS based on statistical significance using the FIMO algorithm and TF binding affinity using the TEPIC algorithm (see Defining transcription factor binding of Methods**)**. The number of TFs, TGs and TFBS corresponding to our three different TFBS identification algorithms (positional/Pos, FIMO, and TEPIC) for both cell lines are also provided in Table 1. After identifying different sets of TFBS, we created corresponding adjacency matrices to generate the motif networks for building the PANDA GRNs. We created binary (binding/no-binding) TF-TG adjacency matrices using the positional and FIMO TFBS. For the TEPIC based adjacency matrix, we used affinity scores of the TEPIC TFBS as weights. We combined these matrices with PPI data and cell type specific co-expression to fit a GRN using the PANDA algorithm (see Generating Gene Regulatory Network Weightings of Methods).

**Table 1:** Number of TFs, TGs and TFBS obtained from different TFBS identification algorithms for GM12878 and K562 cell lines

	GM12878				K562			
	TFs	TGs	TFBS	Unique TF-TG Pairs	TFs	TGs	TFBS	Unique TF-TG Pairs
Pos ChIP-Seq	149	17,106	4,209,133	1,216,272	309	18,190	11,614,248	2,372,274
FIMO	85	16,850	2,444,195	714,167	110	18,173	7,349,429	1,138,823
TEPIC	80	11,784	–	517,226	86	10,239	–	880,554
Promoter	149	11,509	458,959	276,138	308	15,668	1,293,933	681,847
Distal	149	16,964	3,750,174	1,128,079	309	18,152	10,320,315	2,312,490
Intronic	149	17,106	5,896,338	1,378,129	309	18,224	14,764,766	2,820,604

The “Pos ChIP-Seq” row contains TFBS identified by simply extracting the TF peaks in the cis regulatory regions around each gene, “FIMO” row contains statistically significant positional TFBS identified using the FIMO algorithm and the TEPIC row contains positional TFBS extracted based on the TEPIC affinity scores. The remaining rows contain the positional TFBS present within different regulatory elements utilized for the subsequent analyses in the paper. All the ChIP-seq data for the analysis was downloaded from the ENCODE database

After fitting these three GRNs corresponding to the different TFBS identification methods (Pos GRN, FIMO GRN and TEPIC GRN), we constructed corresponding gene expression prediction models using the TF-TG features derived from each PANDA GRN edge-weight set, and a model based on TEPIC affinity scores. Models were constructed using elastic-net based regularized linear regression (ENET) for each cell line. Predictive performance for the models was measured using mean-squared error (MSE), and Pearson’s correlation coefficient (PCC) between predicted and observed expression values of the test set TGs within a fivefold cross-validation framework repeating for 20 iterations (see “Generating training and test data sets for the prediction models” section of the Methods). Each of the PANDA GRNs was generated from *cis* and *trans* TF based regulatory mechanisms, while the TEPIC affinity scores used only *cis*/local regulatory mechanisms, thus providing a direct test of our hypothesis.

**Fig. 2:**
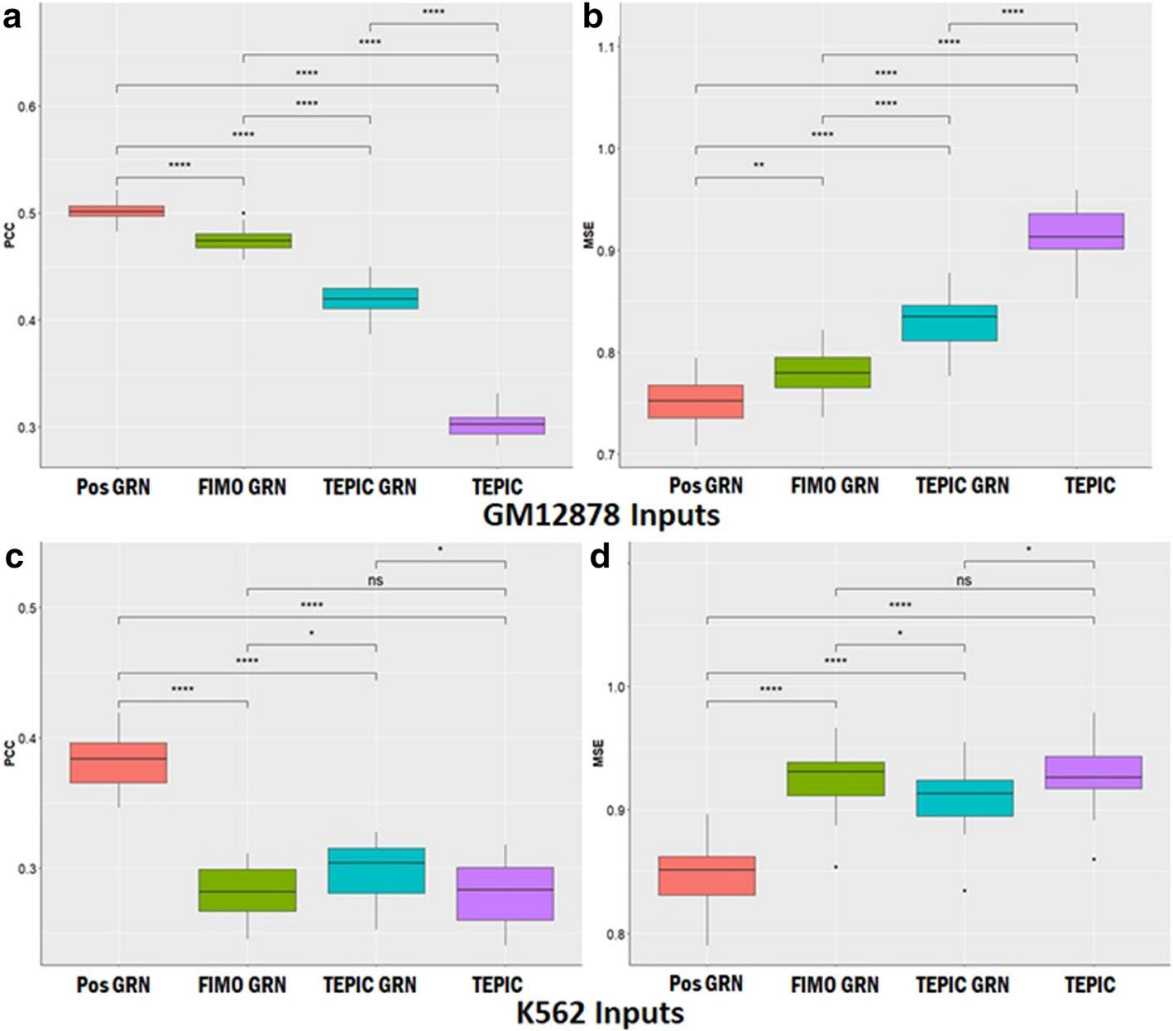
GRN based prediction models outperform those built using TEPIC affinity scores. **a** and **b** correspond to prediction performance for 20 random sets of 1729 GM12878 TGs while C and D were obtained from 1892 K562 TGs. Prediction performances for models corresponding to different inputs were compared using Wilcoxon rank sum test (*** − *p* < 0.0001,** − *p* value < 0.001, * − *p* value < 0.05, ns-not significant)

As shown in Fig. 2, GRN based prediction models containing *cis* and *trans* regulatory mechanisms were more accurate than models built using only *cis*-regulatory TF-TG TEPIC affinity scores. Specifically, the median PCC for TEPIC GRN based models was higher compared to that of TEPIC models for GM12878 (0.42 vs. 0.30, Wilcoxon rank sum test *p* value = 1.45e − 11 Fig. 2a) and K562 (0.30 vs. 0.28, Wilcoxon rank sum test *p* value = 3.50e − 2 Fig. 2c), while the median MSE for the former was lower than that for the latter for GM12878(0.83 vs. 0.91, Wilcoxon rank sum test *p* value = 4.35e − 10 Fig. 2b), and for  K562 (0.91 vs. 0.93, Wilcoxon rank sum test *p* value = 3.26e − 02 Fig. 2d). Results from all analyses (median measures and *p* values) are provided in Additional file 3: Tables S3A–S3I. We also applied the approach to the liver carcinoma cell line HepG2 (Additional File 1), and the results show similar trends.

We also made the following observations from our analyses: (1) Prediction models derived from PANDA GRNs containing biologically relevant CTCF boundary defined *cis*-regulatory TFBS adjacency matrices outperformed the ones built using TFBS derived from a 50Kbp *cis*-regulatory window (Additional file 7: Figure S1). (2) Pos GRN models for GM12878 and K562 had the best performance of all models tested. However, after doing further analyses (Additional File 1), we observed that at least for K562, TEPIC GRN outperformed the Pos GRN models when we used a common set of TF features highlighting the utility of TEPIC in capturing TF-TG regulatory relationships in the form of affinity scores. (3) GM12878 models had the best prediction performance among all the cell lines, which we attributed to the larger sample size (N = 462) utilized for constructing the co-expression network in the PANDA GRN as described in the Additional File 1.

### Expression prediction highlights the regulatory roles of transcription factors

Transcription factors may influence gene expression via a sparse regulatory model where a subset of core TFs have large effects on gene regulation, or via a distributed regulatory model where multiple TFs contribute small collective effects. ENET regression models allow for this heterogeneity by linearly combining two penalizing terms, LASSO (L1) and Ridge (L2), that identify the most influential features (TFs) and shrink the weights of lesser features by either reducing them to 0 (L1), effectively selecting a set of strong factors, by reducing them to a very small number (L2), allowing larger numbers of weak factors in the model. The optimal ratio (α) between these two penalty terms was 0.5 (Additional file 7: Figure S2**),** indicating a balance between a sparse and a distributed regulatory model. This penalizing strategy also helped us in highlighting the correlated functional roles of the TFs (Additional file 1).

**Fig. 3:**
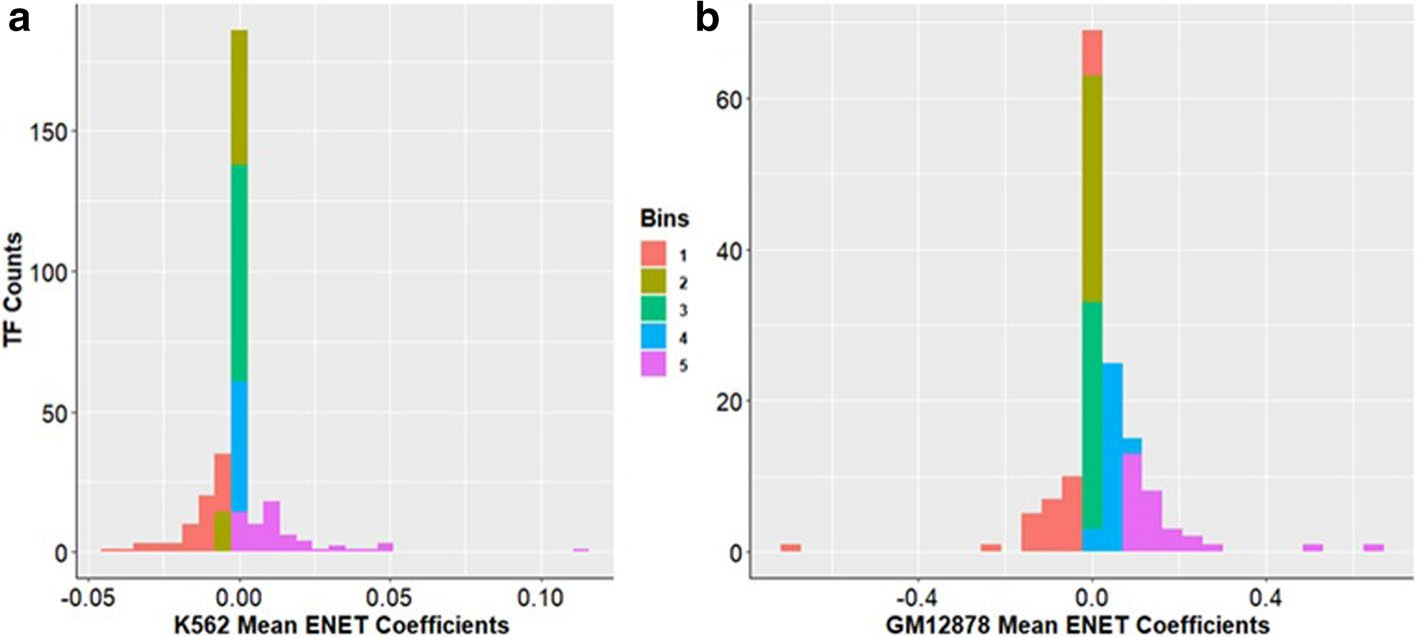
Mean ENET effect estimates reflect the important functional roles of various TFs. Histograms of the average effect estimates for calculated for (**a**) 309 K562 TFs and (**b**) 149 GM12878 TFs 3 using the “Pos GRN” ENET models. We also created 5 bins (quintiles) based on the effect estimates, which are color coded in the histogram

We next averaged the effect estimates of 149 TFs(GM12878) and 309 TFs(K562) from the Pos GRN models fit for 20 iterations using the optimal α of 0.5 (balancing L1 and L2 penalties) and Eq. (1) (see Calculating TF average effect estimates of the Methods). Histograms in Fig. 3 are colored by quintile of these mean effect estimates. We performed a GO enrichment analysis for TFs in each bin and reported the top 5 enrichment terms for biological processes and molecular functions in Additional file 7: Figure S3 for both cell types. We observed that as we moved from positive to negative TF effect coefficients (bin 5 to bin 1), the corresponding GO terms reflect transcriptional activation (bin 5) to those indicating transcriptional repression (bin 1). From this approach, we could derive functions of unannotated TFs based on the bins in which they are placed. For instance, K562 bin 1 contained MYNN(β_K562_ =  − 0.0059) whose function is largely unknown. However, based on its placement in the bin containing strong repressors such as CBX1 (β_K562_ =  − 0.0188), HDAC6 (β_K562_ =  − 0.0045) and BMI1(β_K562_ =  − 0.0341), we predict its function is related to transcriptional repression. Similarly, bin 5 for both K562 and GM12878 contained TFs related to core promoter activity and positive gene expression regulation such as TAF1 (β_GM12878_ = 0.6334), TBP (β_GM12878_ = 0.2142), ELF1 (β_GM12878_ = 0.2249), POLR2A (β_K562_ = 0.1123), POLR2G (β_K562_ = 0.0233), CHD1 (β_K562_ = 0.0492) and MYC (β_GM12878_ = 0.1481). Relatively lesser known TF ZZZ3 (β_GM12878_ = 0.1359; β_K562_ = 0.0375), which was also present in that bin may most likely play a similar transcriptional activation role. We also note that TFs with mean effect estimates very close to or equal to zero were present in bin 2 for GM12878 and in bins 2 and 3 for K562. These TFs were enriched for cofactor activity terms, and their functional annotations reflected their roles as secondary TFs that required binding of the primary TFs to the DNA to exert their influence.

As an additional test of the qualitative impact of including GRN information in our gene expression prediction models, we performed a similar aggregation analysis for the TF effect estimates learned from the TEPIC GRN and the TEPIC only models for the two cell lines, rank-ordering the TFs based on their effect estimates (Additional file 7: Table S2**)**. Compared to the TEPIC only model, we observed an increase in ranks for TFs associated with transcriptional activation as well as a decrease in ranks for the repressive TFs in the TEPIC GRN models. As such, in addition to improving the overall prediction of gene expression, the effect estimates learned from the TEPIC GRN models more accurately represented the GO annotated functional roles of the TFs compared to the TEPIC only models. We provide mean effect estimates for all the TFs for the two cell lines along with their GO enrichment results (Additional file 4: Tables S4A–S4D**)**, and ranks for all the TFs based on their average ENET effect estimates for TEPIC and TEPIC GRN models for the two cell lines (Additional file 5: Tables S5A and S5B).

### Accounting for chromatin interactions between TFBS and gene promoters improves expression prediction

We next examined the impact of TFBS based on the local regulatory context in which they occur. First, we partitioned the TFBS into promoter, intronic, and distal categories (Table 1), and built prediction models using GRNs containing TFBS found only in those regions to assess their predictive performance (see “Additional gene regulatory elements analyses” of the Methods).

**Fig. 4:**
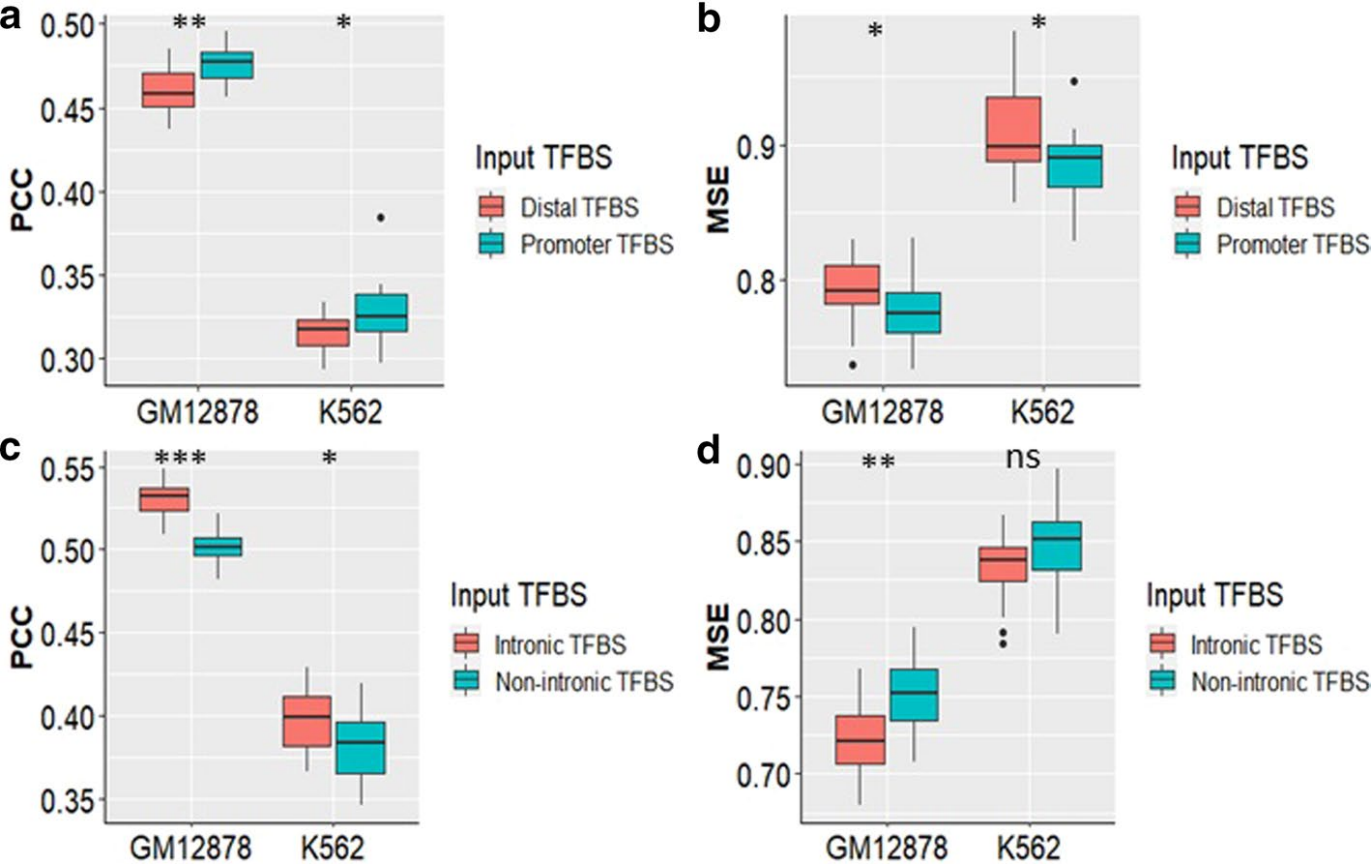
Intronic and Promoter TFBS are important for predicting gene expression. (**a**) PCC and (**b**) MSE obtained from the expression prediction of GM12878 and K56 TGs using models built from GRNs containing promoter and distal TFBS. (**c**) PCC and (**d**) MSE produced by models predicting expression for GM12878 and K562 TGs built using GRNs containing intronic TFBS versus those built without them. The non-intronic TFBS input weights were derived from Pos GRN for both cell types

The promoter region (5 Kb upstream of the TSS of the gene) is important for transcription initiation and regulation and it contains binding sites for pivotal pioneer TFs such as TAFs, POL2 subunits, and TBP. As expected, the median PCC and MSE for the promoter TFBS based ENET models were significantly better than that of the ones containing the distal TFBS alone (Fig. 4a, b) for GM12878 (MSE *p* = 3.26e − 02; PCC *p* = 2.92e − 04), K562 (MSE *p* = 3.75e − 02, PCC *p* = 3.26e − 02). Also, models containing intronic TFBS performed significantly better than those without (Fig. 4c, d) with respect to median MSE (GM12878 *p* = 4.72e − 04) and median PCC (GM12878 *p* = 1.33e − 08; K562 *p* = 2.45e − 02).

**Fig. 5:**
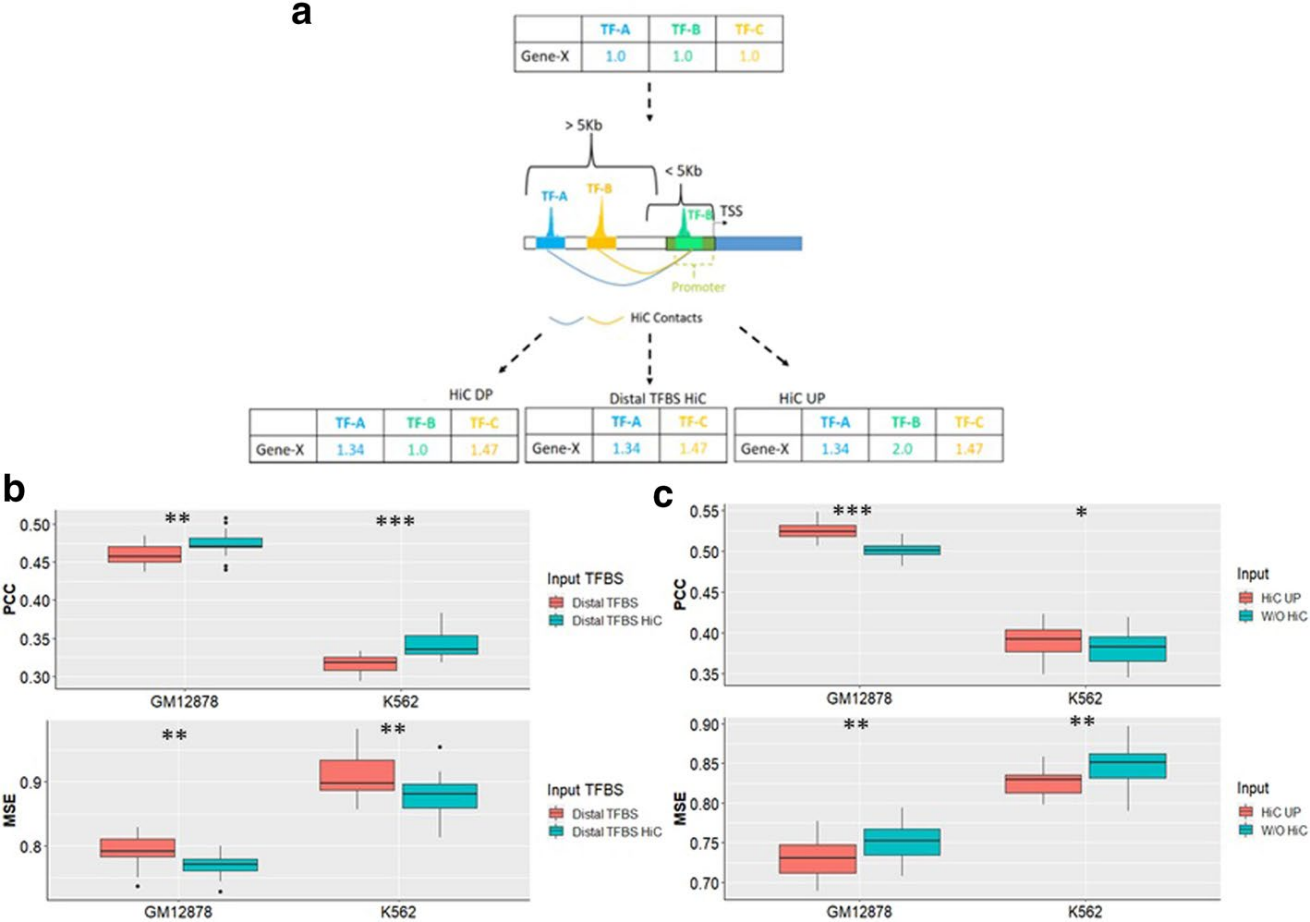
HiC data is capable of capturing the effect of long distance interactions between TF binding within distal TFBS and gene’s promoter on gene expression. (**a**) We used the cell line specific Hi-C data to weight the distal TF-TG interactions in the motif adjacency matrix. We also down-weighted or up-weighted the interactions with the promoter TFs which would have been missed otherwise due to the low resolution nature of Hi-C data. (**b**) We predicted expression of GM12878 and K562 TGs using distal TFBS based GRNs with and without HiC data integration in order to evaluate its predictive value for the models. C shows the predictive performance of the models using GRNs containing HiC normalized motif edges based on the Hi-C UP weighting scheme compared to those built using unweighted binary motif network without HiC information

We next used Hi-C data corresponding to GM12878 and K562 in order to capture long distance interactions between distal TF binding and gene promoters. We used the motif adjacency matrices from the Pos GRN and weighted them based on the number of normalized Hi-C contacts between TF peaks and TG promoters for both cell lines using Eq. (2) as shown in Fig. 5a (see Generating Hi-C Weightings of the Methods). Prediction models including Hi-C adjusted distal TFBS were significantly more accurate compared to the ones built using normal distal TFBS as shown in Fig. 5b with regards to both PCC (GM12878 *p* = 7.33e − 03; K562 *p* = 2.00e − 06) and MSE (GM12878 *p* = 1.43e − 03; K562 *p* = 5.61e − 03) for both cell types.

Next, we expanded this weighting scheme to include promoter TFBS. As promoters are regions of high TFBS activity (as seen in our models, Fig. 4a, b), we expected a high degree of Hi-C contact points within promoter regions. Unexpectedly, these models performed significantly worse; we observed a large number of promoter TFBS (59% for GM12878 and.

90% for K562) that showed no evidence of within-promoter contacts, and using this weighing approach effectively down-weighted promoter TF-TG interactions (Hi-C DP). We therefore also considered an approach that applies the maximum Hi-C weight to all promoter TFBS (Hi-C UP), shown in Fig. 5a. These Hi-C UP based prediction models significantly outperformed all the other models for both cell types as shown in Fig. 5c. These Hi-C UP based prediction models significantly outperformed all the other models for both cell types as shown in Fig. 5c and Additional file 7: Figure S4. Thus, Hi-C data added important regulatory information to our models capturing the effect of long distance interactions between TFs binding to distal regulatory elements and the TG promoter.

### Weighting rare variants using GRN derived effect estimates enriches the SKAT based identification of significant TGs

Determining the impact of rare non-coding variants on TG regulation is a major challenge in the field of human genetics [29]. Here, we demonstrate the utility of the understanding relative TF influence derived from our integrative GRN based prediction framework by weighting rare genetic variants within a kernel-based association test to improve its statistical performance. We used the DGN dataset [27] containing HRC-imputed variant genotypes and RNA-seq from the whole blood of 922 individuals in order to perform SKAT [30] based rare variant analysis. We generated a PANDA GRN for GM12878 based on intronic TFBS motif network weighted using HiC-UP weighting scheme described earlier and then used it to build ENET prediction models and subsequently derived average TF feature weights in the form of effect estimates. We extracted approximately 9.4 million rare SNPs (MAF < 0.01) from the DGN dataset and scored them based on their impact on TF binding intensity using the QBiC-Pred algorithm [31]. By merging this score with the average effect estimates of the corresponding TFs, based on Eqs. (3) and (4), we created a variant scoring metric representing the estimated average effect of a base-pair change on TF-TG regulation in the genome (see QBiC-Pred-GRN rare variant association analysis section of Methods).

**Fig. 6:**
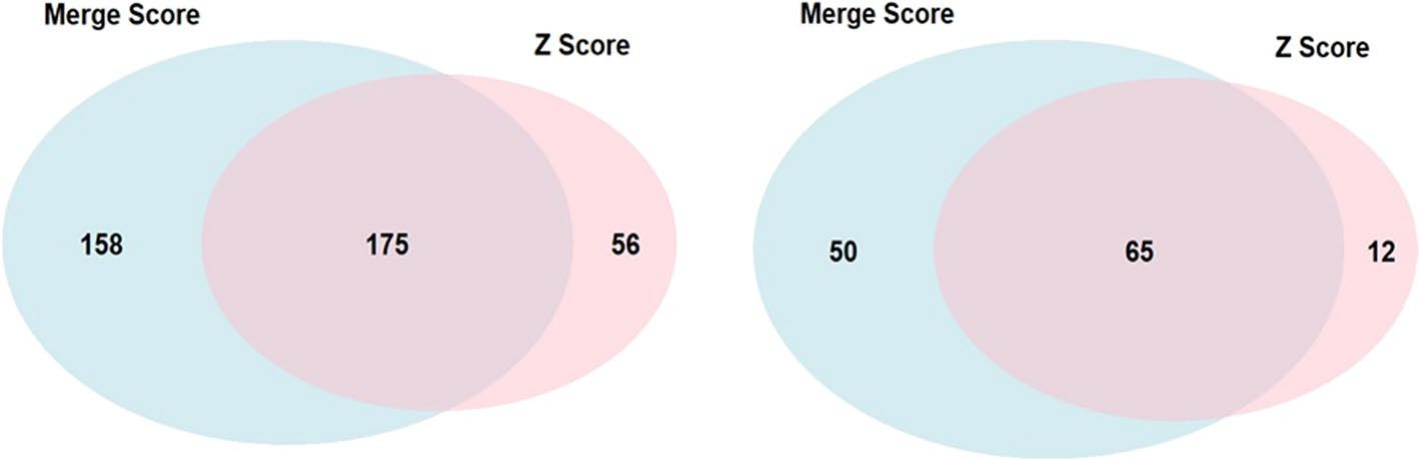
Merging QBiC-pred z-scores with GRN derived TF ENET effect estimates enriches identifications of TGs significantly associated with expression trait. (**a**) shows the venn diagram containing significant TGs (N = 389, *p* value < 4.18e − 06) obtained from the initial discovery analysis based on fitting the merge score and z-score SKAT models using the DGN dataset. (**b**) Shows the significant TGs (N = 127, *p* value < 0.05) identified within the replication analysis done using the GTEx dataset

We used the merged scores to perform SKAT associations to the normalized expression value of TGs in the DGN dataset. We compared the performance of this model to that obtained from aggregated QBiC-Pred z-scores, representing the unweighted effect of rare variants on TF-binding alone. As shown in Fig. 6a, both SKAT models were able to detect 175 common TGs at the multiple hypothesis correction significance threshold of *p* value < 4.18e − 06. Merge score based SKAT model was able to detect 158 unique TGs while z-score based model detected 56 unique TGs at this threshold. We also performed a replication analysis using the whole blood sequencing and expression data from 369 individuals within the GTEx dataset [28]. We were able to replicate 32% of the TGs uniquely identified by merge score based SKAT model (*p* value < 0.05), while only 21% of the TGs uniquely identified by the QBiC-Pred z-score SKAT model replicated (Fig. 6b). Thus, utilizing TF-TG regulatory information learned from our GRN framework for weighting rare variants enriched the identification of TGs, which would have been missed if we had only utilized variant influence over TF binding. We have provided the results from all the SKAT models in Additional file 6: Tables S6A–S6C.

## Discussion

In this study, we developed a modelling framework to predict gene expression within two cellular contexts using gene regulatory networks to capture the *trans* effect of cooperativity and co-regulation on *cis* regulatory factors relative to their TGs. Our models significantly outperformed the ones built using TF-TG affinity scores for *cis*-regulatory features alone by explaining more variance in the TG expression trait.

We further estimated the influence of individual TFs on gene expression outcomes based on their effect coefficients learned from our models. This led to a ranked list of activating and repressive factors influencing transcriptional regulation in both cell lines, including classifications of TFs with previously unknown effects. We observed substantial changes to the ranking of TFs relative to analyses using *cis*-factors alone, illustrating the importance of accounting for the cellular context in interpreting TF effects. While TFs with the strongest and the weakest effects were roughly the same between our baseline TEPIC model and the model overlaid with GRN weights, many TFs with activating and repressive properties show stronger effect estimates after accounting for information captured by the GRN.

As expected, we observed that the highest ranking TFs are crucial for transcriptional initiation and activation, binding within promoter regions of a majority of protein coding genes. The process by which transcriptional machinery forms at the promoter regions of genes has been extensively studied [32]. Promoter TFBS based models were also significantly more accurate at predicting gene expression than models using distal TFBS alone. These results validate our modeling strategy, as these findings are consistent with observations from previous studies [17, 33], and further highlight the important role that promoter regions play in regulating gene expression.

Hi-C data was useful for characterizing long distance interactions between distal TFBS and the gene's promoter. Integrating this data into the PANDA GRNs improved the prediction performance of the models when scaled relative to promoter TFBS. This improvement was also observed in the recently published extension of the TEPIC framework [24].We observed significant improvement in both cell lines despite differences in Hi-C resolution (1 Kb for GM12878 and 5 Kb for K562), however the resolution difference may account for the greater improvement in prediction for GM12878 relative to K562.

Our results also indicate that intronic TFBS provide significant prediction power to the models. There are two likely explanations for this observation. First, introns may bind regulatory TFs or splicing factors that alter the rate of transcription. Previous studies looking at the role of first introns in regulating transcription in *C. elegans* found genome wide occurrence of TFBS in these regions are important in driving gene expression [34, 35]. Second, introns could house alternate promoters for a gene, as noted by analyses of GTEx and FANTOM datasets [36]. For our analyses, we used the upstream TSS of the longest transcript to define gene promoter regions.

Finally, we utilized the TF-TG regulatory information learned from our GRN based framework in order to weight rare variants. This weighting approach led to a significant improvement in power of kernel based SKAT models to detect significant associations with TG expression relative to using weights capturing TF binding affinity alone. While we used linear regression based QBiC-Pred to score TF binding affinity, more complex scoring approaches could also be used within the framework. These analyses demonstrate the utility of our models for annotating otherwise difficult to characterize regulatory variants.

The most direct comparison of predictive performance for our models against published methods is the TEPIC method, which we outperformed. Other approaches have included either more complex modeling techniques or additional histone modification data to improve model performance [15, 17]. Non-linear prediction models such as support vector regression or multi-layer perceptrons applied within our framework may capture more complex interactions among TFs and improve performance. It also remains unclear to what extent the epigenetic context influences the effect a transcription factor has on gene expression. Zhang et al. [15] have demonstrated some redundancy between histone modification and TF binding intensities with respect to gene expression prediction. Thus, inclusion of both histone modification data and TF binding as predictors could diminish the effect of individual TFs, clouding the interpretation of our predictions.

At present, our approach is limited by the availability of ChIP-seq data. Although large scale efforts such as the ENCODE consortium have produced binding data for a large number of TFs in different cell types, this number is still small compared to the actual TFs being expressed in a cell at any given time [37]. This dearth in data availability is due to the difficult and expensive nature of the ChIP-seq experiments themselves [38]. One way to potentially incorporate histone modification and chromatin accessibility data is through the imputation of TF binding not directly measured by ChIP-seq experiments for a given cellular context through techniques like DeepSEA or FactorNet [39, 40]. In future work, these TF binding predictions could supplement the set of inputs to our GRN-based framework to produce better models.

## Conclusions

The modelling approach presented here has multiple applications for studying general factors influencing gene expression. Our models provide an approach for annotating the regulatory structure of a given gene in a tissue or cell-type specific manner, for ranking TFs in order of their likely impact on gene expression, and for clustering genes based on their weighted regulatory features. Our framework also allows for the inclusion of additional functional genomics information, such as higher resolution chromatin interaction data, to evaluate their effect on gene expression. As our understanding of chromatin accessibility and conformation grows, the framework can also be used to better define the *cis*-regulatory window surrounding a gene, which can be useful for eQTL mapping and other downstream analyses. Finally, prioritizing TFs relative to gene expression allows for better prioritization of genetic variants and their influence on nearby gene expression traits. More generally, our approach provides a roadmap for integrating multiple “omics” data sources and assembling fundamental aspects of transcriptional regulation into a coherent portrait of gene expression, which could ultimately help in elucidating mechanisms causing several diseases.

## Methods

All the published algorithms and datasets used in this study have been described in *supplementary data*.

### Defining transcription factor binding sites

We used three methods to define the TFBS between the TFs and the TGs for both the cell types using ChIP-seq data described in Additional file 2: Table S1 and Ensembl gene annotations from GrCh37 human genome assembly:

1. *Positional TFBS* We isolated all the ChIP-Seq peaks within a 50 Kb window upstream of the TSS of the longest transcript and downstream of the body of each protein coding TG. We then used the most distant CTCF peaks to demarcate the *cis*-regulatory boundaries for these TFBS, as it is a well-known insulator protecting the enhancers of TG gene from acting upon the promoters of another as shown in Fig. 1 [41].
2. *FIMO TFBS* We applied the FIMO algorithm [42] from the latest release of the MEME-suite tools(*v.5.1.1*) on the “Positional TFBS” data to find statistically significant set of TFBS. We extracted genomic sequence underneath the TF peak corresponding to each TFBS and the JASPAR(*v.2020*) based TF position weight matrices(PWM) to find statistically significant TFBS at the *p* value threshold of 0.01.
3. *TEPIC TFBS* We downloaded the TEPIC software (https://github.com/SchulzLab/TEPIC) along with the position specific energy matrices(PSEMS) for all TFs [16]. We used these PSEMS, the Ensembl Homo_sapiens.GRCh37.87.gtf annotation, and our predefined Positional TFBS to find affinity scores for TFs binding in the 50 Kb window around each TG’s TSS.

### Generating gene regulatory network weightings

We converted the unique TF-TG interactions obtained from each TFBS identification method into weighted (TEPIC) and unweighted (Pos ChIP-Seq and FIMO) adjacency matrices. We used these matrices, along with BioGrid (*v.3.5.188*) [43], a method for defining protein–protein interactions (PPI), and cell-type specific co-expression networks to generate three different PANDA outputs. After 25 iterations, we obtained convergence by setting the threshold for Hamming’s distance at 0.001 and by using the value of 0.1 for the update parameter for each GRN.

### Generating training and test data sets for the prediction models

We used four different input datasets, for each cell type, for our prediction models based on PANDA GRN edgeweights (“Pos GRN”, “FIMO GRN”, “TEPIC GRN”) and TEPIC affinity scores (“TEPIC”) as shown in Fig. 1*.* Using these matrices as inputs, we predicted the expression for independent datasets of GM12878 (ENCSR889TRN) and K562 (ENCSR545DKY) using the linear regularized elastic net(ENET) regression models. We used the python-based implementation of the ENET model from the *scikit-learn* library to build the prediction models, setting the value of $\alpha$ (the ratio between the lasso and ridge norms) at 0.5.

We used the log10-normalized FPKM (fragments per kilobase of transcripts per million) for TGs, that were common among different input matrices described in Table 1 and also contained promoter Hi-C contacts with distal TFBS, as the response vector for the ENET prediction models. Thus, the models contained 8,644 TGs for GM12878, and 9460 TGs for K562. We also applied our approach to 12,013 TGs for HepG2 for additional validation and generalization.

We split the input feature matrix and the output expression vector into 80% training data and 20% test data. We used the training data to train the ENET models, using 20-fold inner cross validation. We then predicted the expression of the test set genes, using the learned ENET models and calculated mean squared error (MSE) and Pearson’s correlation coefficient (PCC) to measure the predictive performance for the models. We repeated this process for 20 iterations as shown in Fig. 1.

### Calculating TF average effect estimates

We calculated the average effect estimate for TF *T* $\overline{\beta }_{T}$ using the following equation:

1 $$\overline{\beta }_{T} = \frac{1}{\left| N \right|}\mathop \sum \limits_{n\epsilon N} \beta_{T,n}$$

Here $N$ is the set of random instances that we used to build our prediction models and $\beta_{T,n}$ is the effect estimate of *T* for instance $n$. We only used the GM12878 and K562 Pos GRN prediction models in order to calculate these estimates. We further divided the TFs based on these mean effect estimates using the *xtile* function of R(*v.3.4.2*) into 5 roughly equal bins.

### Additional gene regulatory elements analyses

We generated additional TFBS datasets by extracting TF peaks overlapping TG intronic regions, promoter regions (5 Kb upstream of the TSS) as well the ones present in distal region beyond the promoter (Additional file 7: Figure S5A). The number of corresponding TFBS and TF-TG interactions for each cell-type representing these regions is provided in Table 1. In order to get the intronic regions for each TG, we first obtained the exonic regions corresponding to all the transcripts for a given TG and then subtracted them from the regions spanning the respective transcript lengths using *bedtools* (Additional file 7: Figure S5B). We added the TFBS present in the intronic regions to the positional ChIP-Seq TFBS dataset to create the intronic TFBS dataset for each cell line. We used TF-TG interactions based on these additional TFBS datasets to create motif-based adjacency matrices and used them to build additional PANDA GRNs, which we ultimately used to predict gene expression for TGs common between the models we were comparing.

### Generating Hi-C weightings

We accessed Hi-C data for K562 (GSM1551620) with 5 Kb resolution and for GM12878 (GSM1551688) with 1 Kb resolution. We defined the promoter as the 5 Kb region upstream of the TSS of the longest transcript for each gene. We normalized the Hi-C interactions using the Knight Ruiz (KR) normalization and created sparse contact matrices for both cell types. We calculated the number of contact points between each TF peak within a gene’s distal regulatory region and its promoter using *bedtools v.2.27.1*. We then calculated the HiC adjusted edge-weights between each TF and TG using the following formula:

2 $$C_{i,g} = 1 + scaled\left( {\frac{1}{{N_{i,g} }}\mathop \sum \limits_{{p\epsilon P_{i,g} }} c_{p} } \right)$$

Here $C_{i,g}$ is the Hi-C adjusted edge weight between TF $i$ and TG $g$, $N_{i,g}$ is the number of ChIP-seq peaks corresponding to $i$ in the regulatory region of $g$, $P_{i,g}$ is the set of peaks corresponding to $i$ in the regulatory region of $g$ and $c_{p}$ is the number of KR normalized contacts made by peak $p$ with the promoter of $g$. We used the MinMax scaling function of the *scikit-learn* library to scale the mean contacts within the (0, 0.99) range. Thus, if the TF did not contain any peaks interacting with a gene’s promoter, the $C_{i,g}$ would be equal to 1 and the maximum value for $C_{i,g}$ would be 1.99. We generated the cell type specific “Hi-C DP” motif adjacency matrix using these scaled interactions. We then extracted all the promoter-based TF-TG interactions that were down-weighted to 1.0, or were found to have no Hi-C interactions, in the “Hi-C DP” matrix and gave them maximum weight of 2.0 to create the cell-type specific “Hi-C UP” adjacency matrix. We created two new GRNs using these adjacency matrices as motif networks along with the cell-type specific PPI and co-expression data to build prediction models following the workflow described in Fig. 1.

### QBiC-Pred-GRN rare variant association analysis

**Fig. 7:**
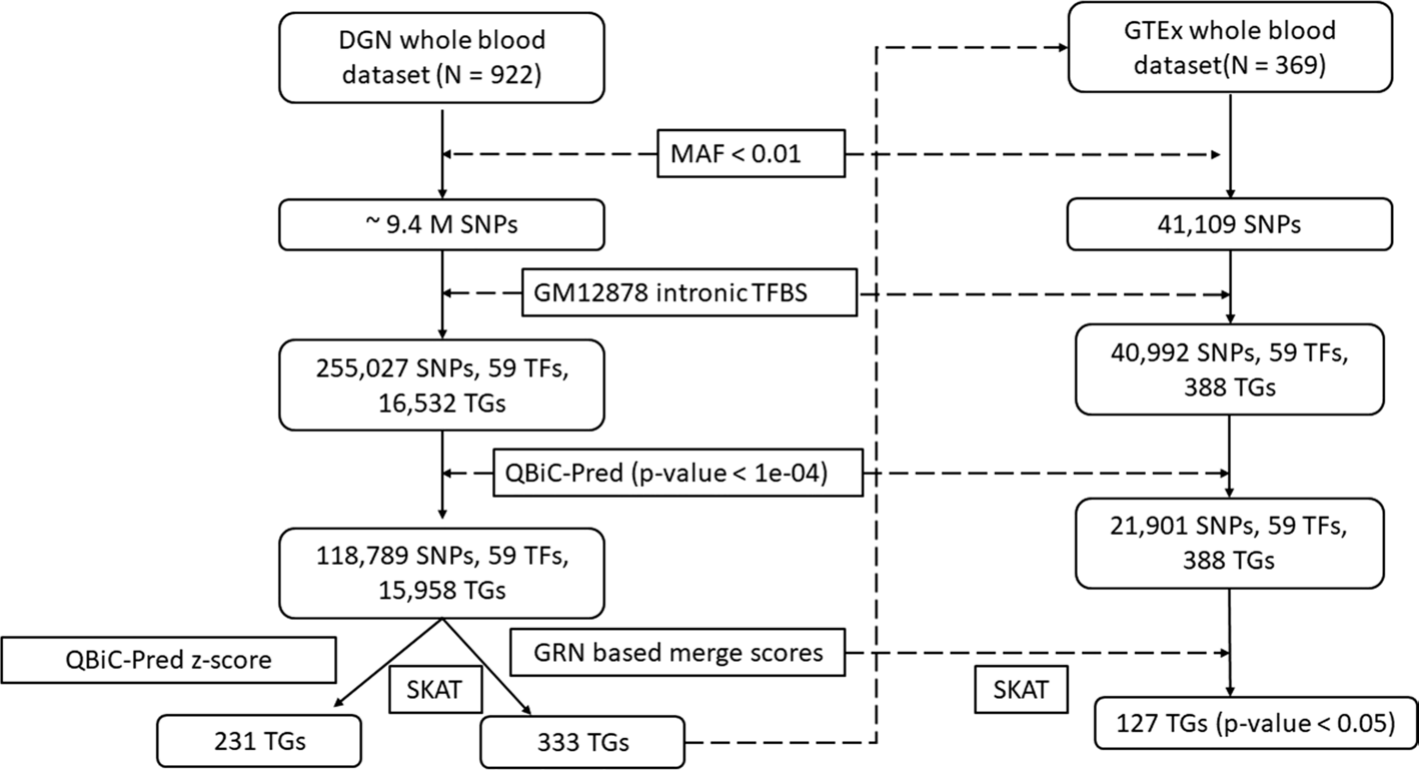
The workflow of our rare variant analysis. We used the DGN dataset for initial discovery analysis and the GTEx dataset for the replication analysis

We followed the workflow shown in Fig. 7 for the rare variant analysis. We generated GM12878 GRN utilizing the intronic TFBS for motif network and HiC up weighting scheme described previously. We then fit the ENET models using TF-TG edgeweight features from this GRN, and used the learned models to compute average TF effect estimates based on Eq. (1). For the initial discovery analysis, we used the depression genes and networks (DGN) data set, which contains genotypes and RNA-seq data for 922 individuals of European descent [27].We further imputed variant genotypes using 1000 genomes reference panel and the University of Michigan imputation server [44, 45]. We extracted rare variants at a minor allele frequency (MAF) threshold of 1% (N ≈ 9.4 M variants) and overlapped them with the GM12878 intronic TFBS.

Out of the 149 TFs, we were able to find trained QBiC-Pred models for 59 TFs. We scored these variants using the offline version of the QBiC-Pred software [31] which we downloaded from the github repository (https://github.com/vincentiusmartin/QBiC-Pred). We used the *p* value threshold of 0.0001 to identify the variants significantly impacting the TFBS we identified 118,789 rare variants that were present within their binding sites.

We merged the z-score obtained from the QBiC-Pred algorithm and the TF effect estimates for each rare variant present within the TFBS for each TG using the following sets of equations.

3 $$Z_{{v,t,g}} = ~\overline{{\beta _{t} }} \times \frac{{\mathop \sum \nolimits_{{p_{{t,g}} \in P_{{T,g}} }} z_{{v,p_{{t,g}} }} }}{{\left| {P_{{t,g}} } \right|}}$$

4 $$S_{v,g} = \frac{{\mathop \sum \nolimits_{{t \in T_{g} }} Z_{v,t,g} }}{{\left| {T_{g} } \right|}}$$

Here $z_{{v,p_{t,g} }}$ is the QBiC-Pred z-score for variant $v$ significantly impacting the peak region(TFBS) $p_{t,g}$, which is a subset of all the peak regions $P_{t,g} \;{\text{belonging}}\;{\text{to}}$ TF $t$ within the regulatory/intronic regions of TG $g$. $\overline{{\beta_{t} }}$ is the average ENET effect estimate obtained from the learned ENET models for TF $t$ and $Z_{v,t,g}$ is the scaled QBiC-Pred z-score for variant $v$ corresponding to TF $t$ binding *cis-*regulatory/intronic regions for TG $g$. $S_{v,g}$ is the merge score for variant $v$ for each TG $g$ computed by averaging the scaled z-scores for all the TFs present within the *cis*-regulatory/intronic regions of TF $g$($T_{g}$). We also computed aggregate QBiC-Pred z-scores for each variant present within all the TFBS for each TG $g$ without utilizing the average effect estimates. In other words, we simply removed the effect estimate ($\overline{{\beta_{t} }}$) from the set of equations described above. We scaled both aggregated z-scores and merge scores within the range [− 1,1] and used them for weighting the variants.

We used the R implementation of the SKAT algorithm [30] (*v 2.0.0*) in order to find association between these sets of variants and the TG expression levels normalized by HCP(hidden covariates prior). We used the merge scores and QBiC-Pred aggregated z-scores as variant weights for the SKAT kernel matrices and fit the models for 11,650 TGs using 74 additional biological and technical covariates provided within the DGN dataset.

For replication analysis, we utilized the Genotype-Tissue Expression(GTEx) dataset containing whole genome sequencing and RNA-seq data for 369 individuals [28] (Fig. 7). We repeated the analysis done for the DGN dataset to extract and score variants and then performed SKAT using the normalized expression of TGs that were found significant in the DGN analysis and whose expression values were present in the GTEx dataset (N = 388). For GTEx analysis, we utilized the 65 covariates provided within the dataset to fit the SKAT model.

### Statistical evaluations

We used R *v.3.4.2* to perform all the statistical analyses in our study. Assuming a non-normal distribution of the PCC and MSE produced by the prediction models, we used the Wilcoxon rank sum test to compare medians of these performance measures for different models. We used the *gseapy* package in python for gene ontology (GO) enrichment analyses. We divided the TFs into 5 bins (quintiles) based on their average effect estimates and ran the enrichment analysis for GO biological processes (GO BP) and GO molecular functions (GO MF) terms using all cell-type specific TFs as background. We specifically looked for significant enrichment terms (adjusted *p* value < 0.05) for each bin for both the GO categories. We then extracted the top 5 significant enrichment terms for each bin (provided in Additional file 4: Tables S4C and S4D) based on their *p* values and plotted them in Additional file 7: Figure S3.

## Supplementary Information

**Additional file 1.** Supplementary information.

**Additional file 2: Additional Supplemental File**. This file contains additional supplementary information that is not directly relevant to the conclusions drawn in the paper, but should be interesting for the readers to look at.

**Additional file 3: Supplementary Table S1.** Encode accessions for the TF ChIP-Seq files used in the paper.

**Additional file 4: Supplementary Table S3.** Prediction results from fitting the ENET models using different input features.

**Additional file 5: Supplementary Table S4.** Results corresponding to TF effect estimates and the GO enrichment analysis based on these effects.

**Additional file 6: Supplementary Table S5.** Results from comparing the TF effect estimates obtained from the TEPIC GRN and the TEPIC models for GM12878 and K562 cell lines.

**Additional file 7: Supplementary Table S6.** Results from fitting the SKAT models.

## Data Availability

We have created a docker container as well as a github repository with all the data files and scripts used for analysis. (https://hub.docker.com/orgs/bushlab/repositories/centos_tf_grn, https://github.com/bushlab-genomics/TF_GRN) Bio-samples and/or data for this publication were obtained from NIMH Repository & Genomics Resource, a centralized national biorepository for genetic studies of psychiatric disorders. The GTEx dataset used for the analyses described in this manuscript were obtained from dbGaP at http://www.ncbi.nlm.nih.gov/gap through dbGaP accession number phs000424.v8.p2.

## Abbreviations

TF: Transcription factors
TG: Target gene
ChIP-Seq: Chromatin immunoprecipitation sequencing
PANDA: Passing attributes between networks for data assimilation
GRN: Gene regulatory network
TFBS: Transcription factor binding site/s
FIMO: Find individual motif occurences
PWM: Position weight matrix
ENET: Elasticnet
FPKM: Fragments per kilobase of transcripts per million
GO: Gene ontology
PCC: Pearsons correlation coefficient
MSE: Mean squared error
QBiC-Pred: Quantitative TF binding change predictions due to sequence variants
eQTL: Expression quantitative trait loci
SKAT: Sequence kernel association test
DGN: Depression genes and networks
SNP: Single nucleotide polymorphism
GTEx: Genotype-tissue expression
GO MF: Gene ontology molecular function
GO BP: Gene ontology biological process
PSEMS: Position specific energy matrices
TSS: Transcription start site
PPI: Protein–protein interactions
ENCODE: Encyclopedia of DNA elements
IDR: Irreproducible discovery rate
