# Supplementary material for: Modeling transcriptional regulation using gene regulatory networks based on multi-omics data sources
Source: BMC Bioinformatics. 2021 Apr 19;22:200. doi: 10.1186/s12859-021-04126-3 (PMC8056605; doi:10.1186/s12859-021-04126-3)
Supplement: Supplementary file 1 — Additional file 1. Supplementary information. [file 12859_2021_4126_MOESM1_ESM.docx]

**Modeling transcriptional regulation using gene regulatory networks based on multi-omics data sources**

Neel Patel^1,2^ and William S. Bush^2^

^1^Department of Nutrition, Case Western Reserve University, Cleveland, OH, USA. ^2^Department of Population and Quantitative Health Sciences, Case Western Reserve University, Cleveland, OH, USA.

**Additional Supplemental Information**

**Additional supplemental methods**

**Identifying literature annotated TF-TG interactions**

We downloaded TF-TG interactions present in the Harmonizome[1] and TRRUST (version 2.0)[2]. From the Harmonizome database, we downloaded interactions corresponding to “JASPAR Predicted”, “ENCODE”, “CHEA”, “TRANSFAC”, “TRANSFAC Predicted” and “MotifMap Predicted” datasets. These datasets contained TF-TG interactions identified by using motif based computational analysis and literature based annotations. On the other hand, the TRRUST database contains TF-TG interactions defined using sentence based text mining approach by analyzing over 20 million PubMed articles. We then overlapped these interactions with the PANDA GRN edges for all the three cell-lines.

**Computing correlation matrix using PANDA GRN based TF features and performing k-means clustering.**

Using the “Pos GRN” TF-TG features for each cell-line, we created symmetric correlation matrices for the TFs based on Pearson’s correlation coefficient. Using these correlation matrices, we performed k-means clustering to define 5 clusters for each cell-line. We used the “kmeans()” function in R(version 3.4.2) in order to perform this analysis. We plotted these clusters using the “fviz_cluster()” function from the “factoextra(v. 1.0.7)” R package.

**Results from the GM12878 and K562 cell lines generalized to HepG2 as well.**

**Additional Table 1: The following table represents the number of TFs, TGs , TFBS and TF-TG interactions used for each input dataset in the HepG2 analysis.**


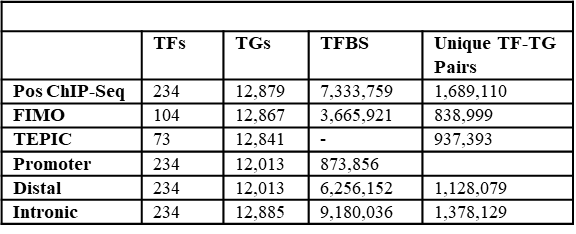

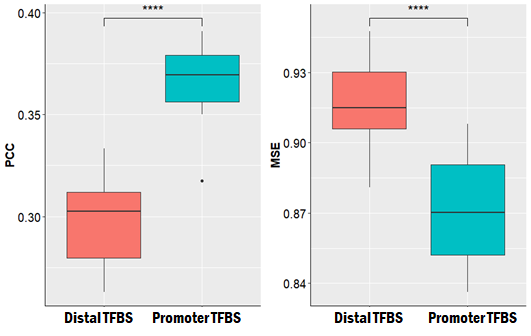

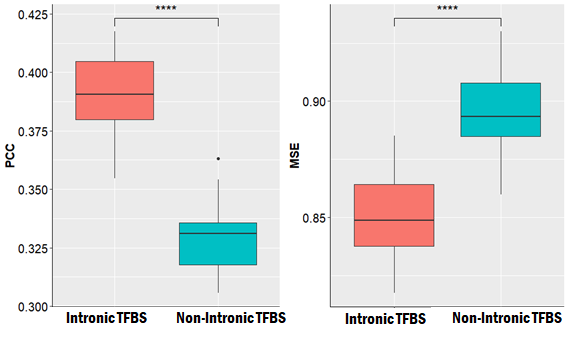

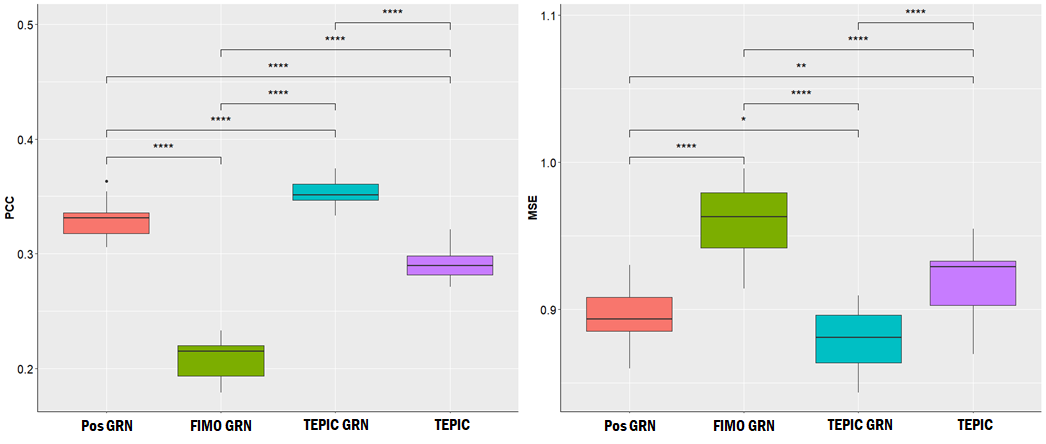


**A**

**B**

**C**

**D**

**Additional Figure 1: HepG2 analysis. We downloaded ENCODE data corresponding to 234 HepG2 TFs and found TFBS for 12,887 TGs. A) After generating GRNs based on positional ChIP-seq data, FIMO based statistically significant TFBS and calculating TEPIC scores, we compared prediction performance of the corresponding ENET models. B) MSE for the above models. Boxplots showing the comparison of the intronic vs. non-intronic TFBS based prediction model performance in C and of distal vs. promoter TFBS based prediction models in D**

**The higher number of features in the GM12878 and K562 led to better prediction performance.**

As shown in **Figure 2** of the main text, Pos GRN models for GM12878 and K562 had the best prediction performance among all the models built using different TFBS identification algorithms. This could be due to the overfitting caused by the highest number of TF features in these models. To test this, we restricted the analysis to GRNs built using the same number of TFs. The performance of Pos GRN in this restricted analysis was similar to that of FIMO GRN for all cell-lines (**Additional Figure 2**). Furthermore, in this sensitivity analysis we found that TEPIC GRN-based models were the most accurate for K562 and HepG2. In other words, when restricted a common set of TFs, TEPIC affinity scores were able to capture more regulatory information between TFs and TGs in comparison to simple positional ChIP-seq data and statistically defined FIMO-based TFBS for K562 and HepG2. prediction models are provided in the **Supplementary Tables S3A-S3I**.


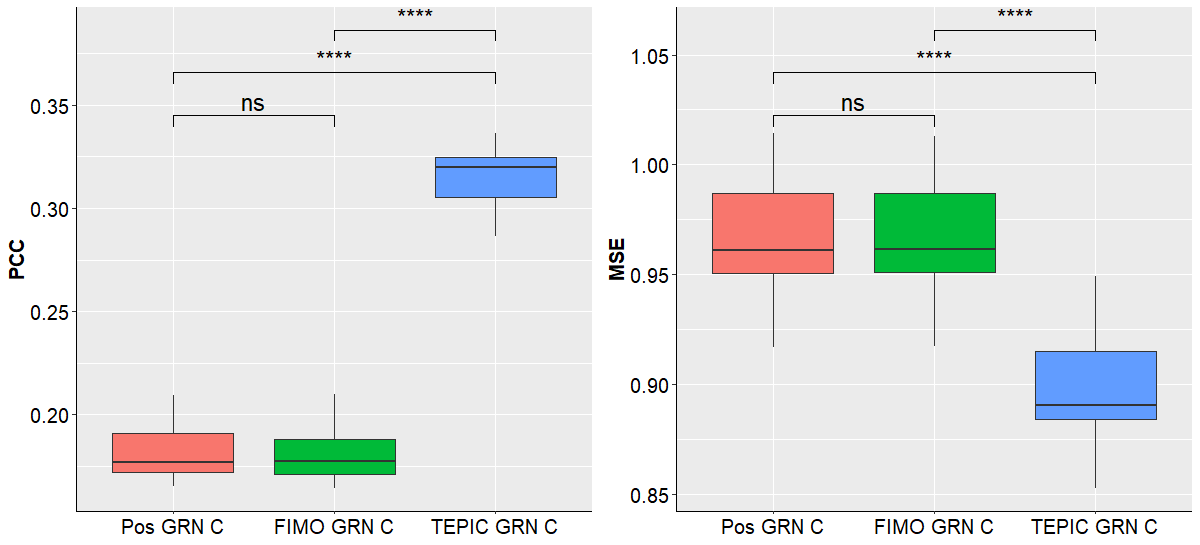

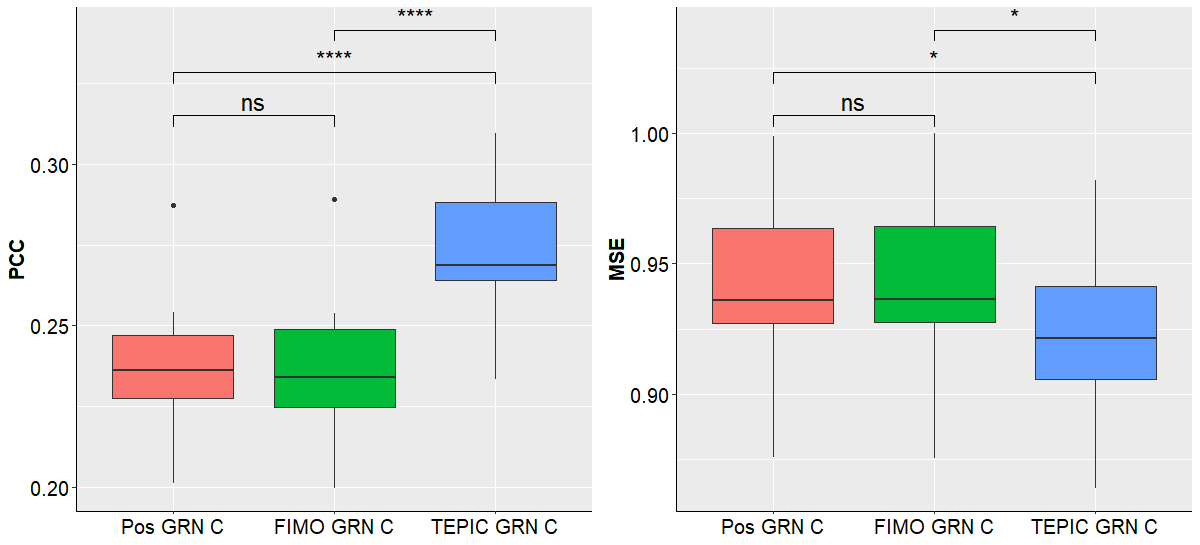

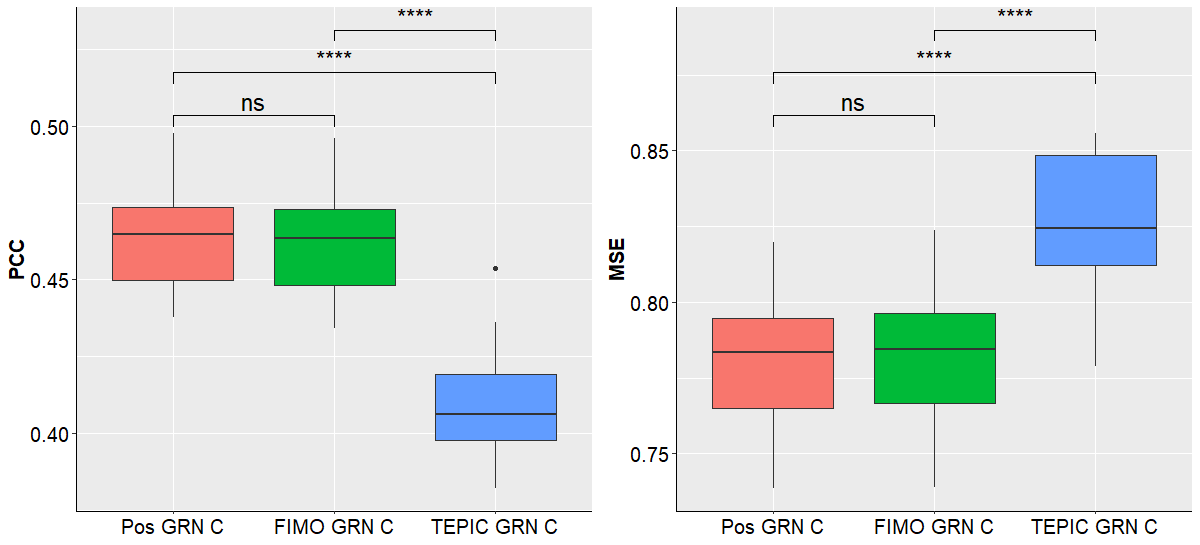


**HepG2 Inputs**

**K562 Inputs**

**GM12878 Inputs**

**Additional Figure 2: The results from fitting prediction models with the same number of TF features for the inputs shown in Figure 1(main text). The box plots are showing results from models containing 77 GM12878 TF features(A), 86 K562 TFs(B) and 73 HepG2 TFs(C). Here, we have added “C” in order to represent the comparable models and differentiate them from those shown in Figure-1**

**A**

**B**

**C**

**GM12878 models had the prediction performance of all the cell-lines.**

As described in the main text, the GM12878 based models were the most accurate out of the three cell-lines for predicting TG expression. In order to consolidate for the difference in the number of TFs among the cell-lines, we utilized a common set of 61 TFs among the 3 cell-lines as well as a set of 110 TFs common between GM12878 and K562 to generate the PANDA GRNs and subsequently predict TG expression. As shown in **Additional Figures 3A** and **3B**, even in this restricted analysis, the performance of the GM12878 based models was significantly superior both for the 3 cell comparison( PCC_GM12878_ = 0.32, PCC_K562_=0.22, PCC_HepG2_=0.20) as well as for the 2 cell comparison(PCC_GM12878_ = 0.37, PCC_K562_ = 0.24). We also assessed the performance of the GM12878 GRN models for predicting cross-cell type TG expression. For this analysis, we tested the prediction performance of the models trained using regulatory information from one cell-line on the test genes of the other cell-lines. As shown in **Additional Figures 3C** and **3D**, using TFs and TGs common between GM12878 and K562, we were not able to obtain decent prediction performance as shown in. However, the GM12878 Pos GRN based model performed better for K562 TGs than vice-versa. Similarly, GM12878 produced median PCC of 0.17 for K562 cell-line for the pairwise comparison and that of 0.14 for the three cell type comparison. On the other hand, despite being derived from a different lineage, the HepG2 cell-type produced decent median PCC of 0.12 for the GM12878 TGs but the prediction performance was very poor for the K562 TGs (Median PCC = -0.014). K562 based GRNs did not produce good cross-cell type prediction performance in both the pairwise and the three way comparisons. Thus, GM12878 GRN based models had the best within cell-type as well as cross cell-type prediction performance. We hypothesized that this stark difference in the prediction performance was due to the markedly smaller sample size of the PANDA expression data for K562 and HepG2 relative to GM12878 (9 and 8 vs 462). In order to detect the impact of the co-expression dataset on the prediction performance of the models, we eliminated PPI and co-expression datasets individually and together from the GRN and replaced them with an identity matrix. As shown in **Additional Figure 4**, the prediction performance for the GRN containing just the TFBS based motif information was the poorest for all the cell-lines. This was expected as this GRN was devoid of the information from other regulatory mechanisms. Moreover, the GRN containing motif and PPI information produced worse prediction models compared to the ones containing motif and co-expression datasets for the three cell-lines. The performance of “M+E” GRN was comparable to the one containing all the three types of networks, and that of “M+P” was comparable to the one containing just motif information. Thus, we were able to conclude that the co-expression datasets provided important information to generate PANDA GRN as they captured the correlation patterns for genes that were co-regulated by the same set of TFs.

Lastly, we also explored the accuracy of the TF-TG regulatory information captured by the Pos GRNs corresponding to the three cell-types. We downloaded the TF-TG interactions defined within the Harmonizome and the TRRUST(v.2.0) datasets based on literature annotations and motif based computational predictions. We overlapped the PANDA GRN based TF-TG edges on top of these interactions and plotted them, along with their edge-weights, in the histograms shown in **Additional Figure 5**. For the GM12878 cell-line, we observed that 527,636 (43%) PANDA edges were present within the annotated TF-TG interactions, while for K562 and HepG2 the number of edges present within the annotated TF-TG interactions were 567,325(24%) and 443,080(26%) respectively. The higher number of literature annotated TF-TG interactions for the GM12878 GRN could be due to the more extensive co-expression dataset used to build the network leading to a more accurate estimation of TF-TG regulatory relationships compared to the other two cell-lines.

**Additional Figure 3: Boxplots showing the prediction performance of GRNs generated using information corresponding to A) 61 TFs commons among GM12878, K562 and HepG2 and B) those generated using 110 TFs common between GM12878 and K562. C and D show the correlation plot for the cross-cell-type prediction performance for these models.**


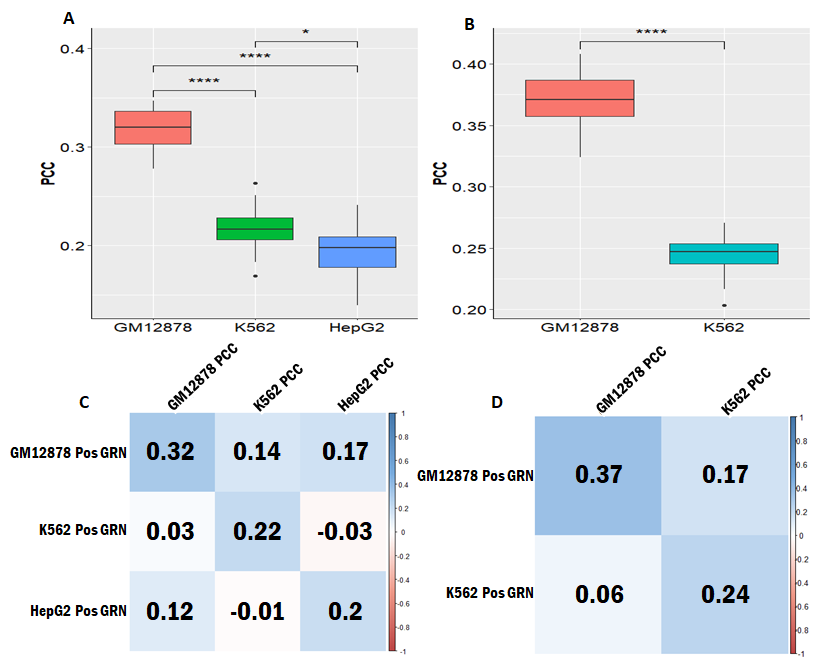

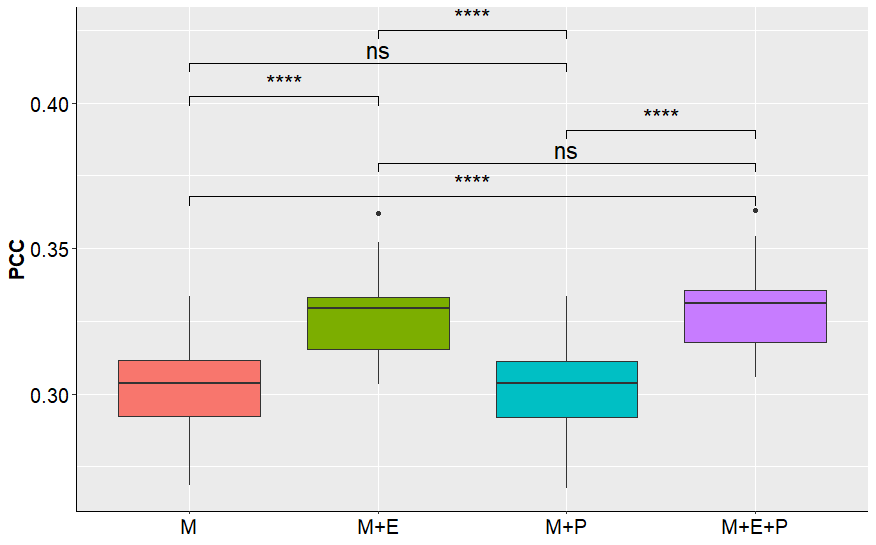

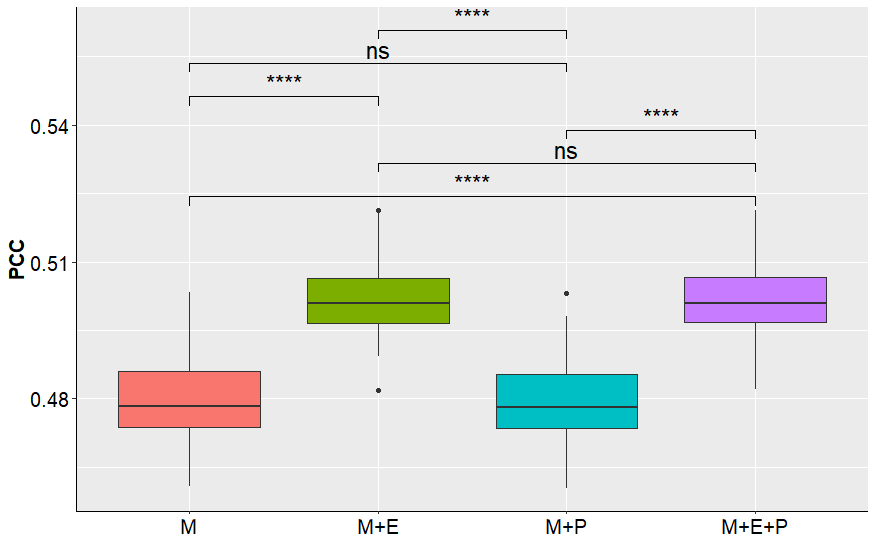

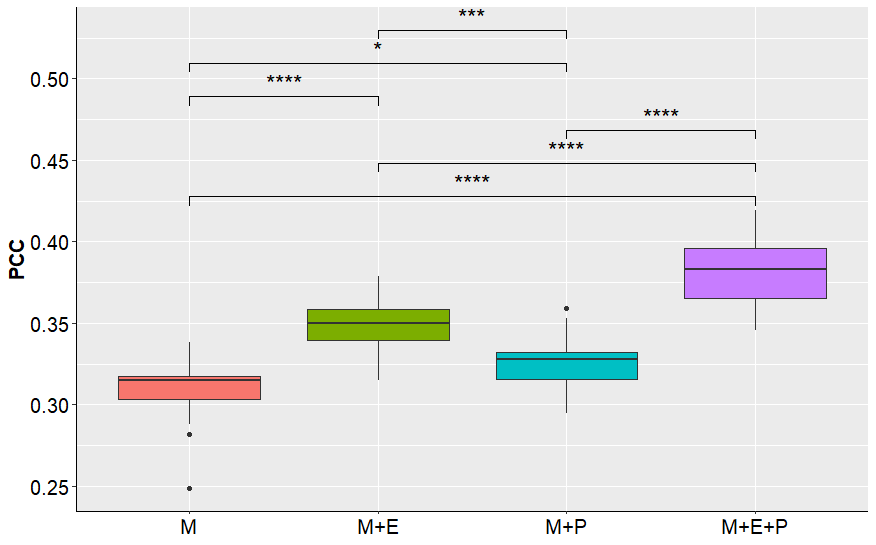


**GM12878**

**K562**

**HepG2**

**C**

**B**

**A**

**Additional Figure 4: Impact of removing different datasets from the PANDA GRN on prediction performance. “M” represents GRN containing Motif network, “M+E” represents one containing Motif and Co-expression datasets; “M+P” represents one containing Motif and PPI datasets and finally “M+E+P” represents the GRN containing all three datasets. The boxplots were created from the PCC obtained from predicting expression for 20 instances of A)1895 K562 B)1751 GM12878 and C) 2403 HepG2 test genes.**


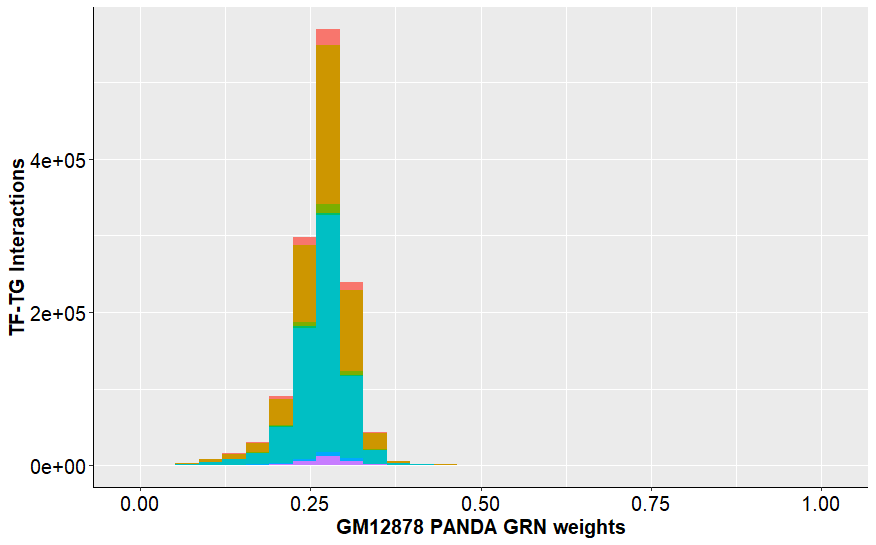

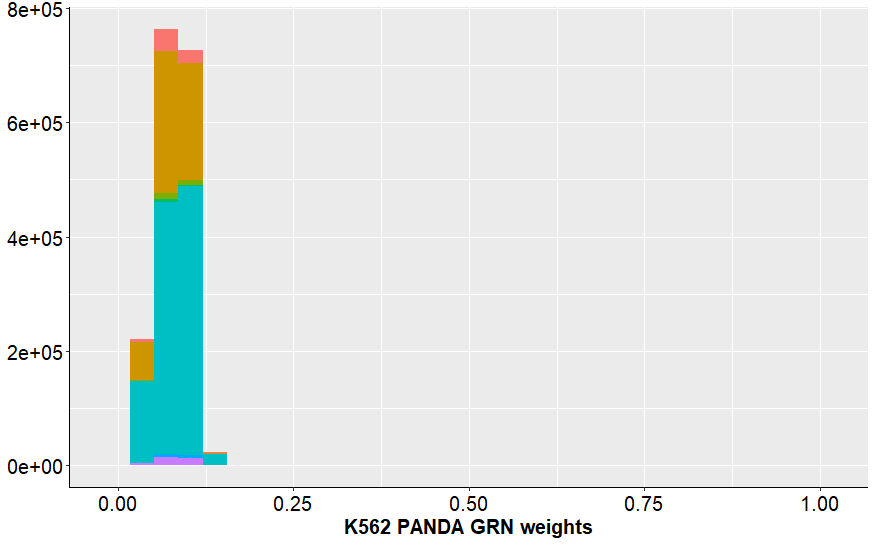


**A**

**B**

**Additional Figure 5: Significant proportion of the edges present in the PANDA GRN networks represented known and predicted TF-TG interactions. We looked for annotated and predicted TF-TG interactions present in the Harmonizome and TRRUST databases corresponding to the edges in the PANDA GRNs for the three cell-lines. Histograms in the figure show the scaled Pos GRN edge-weights for the unique TF-TG interactions binned according to the annotation dataset shown in the legend for A) GM12878, B) K562 and C) HepG2 cell-lines.**


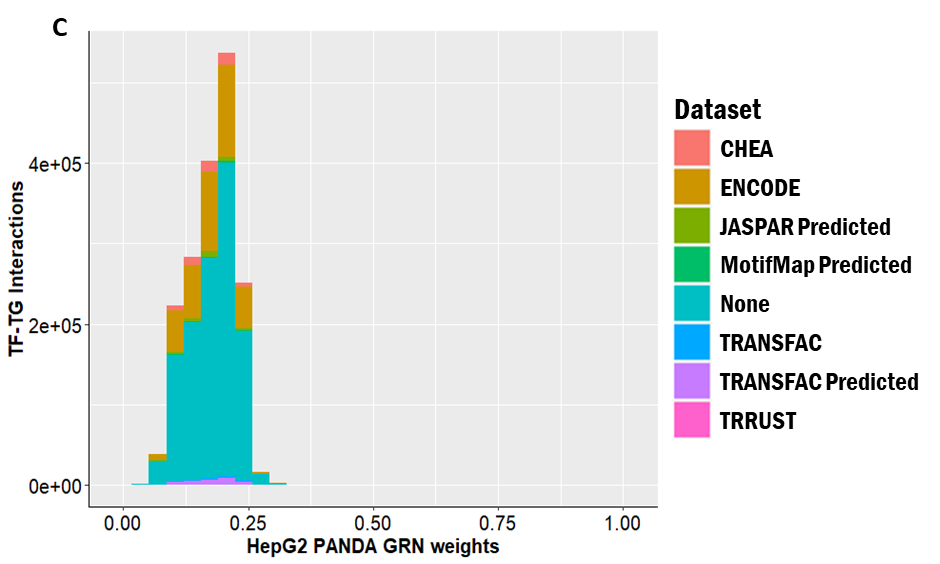


**ENET models were able to capture the correlation structure within TF features as defined by the PANDA GRNs**.

ENET models use LASSO and ridge penalty terms to shrink effect estimates corresponding to features that don’t contribute towards explaining the variance in the outcome[3]. This shrinkage of weights helps in reducing a complex model into a simple one and in overcoming the multi-collinearity within features. Using the Pos GRN ENET models learned from predicting the expression of TGs for K562 and GM12878 cell-lines, we calculated the average effect estimates for each one of the TFs. Of the 149 GM12878 TFs, 45(30%) had really small effect estimates in the range [-0.01,0.01], while for the 309 K562 TFs, 173 (56%) had their weights shrunk to really small size. In order to determine the effect of the correlation structure of the PANDA GRN feature weights on this shrinkage, we performed k-means clustering to define 5 clusters based on the correlation matrix derived from these weights. These clusters shown in **Additional Figures** **6A** and **6C** contained TFs with highly correlated GRN based feature weights. For each cluster, we plotted the corresponding mean effect estimates shown in the histograms in **Additional Figures** **6B** and **6D**. These histograms represent the mean ENET effect estimates in relation to the correlation structure within the TF features for the two cell-lines. Each cluster can be seen containing some TFs with high effect estimates in both directions, while some had effect estimates close to zero. For instance, cluster 5 in GM12878 contained 63 TFs, of which 26 had mean effect estimates in the range [-0.01,0.01], while estimates for 16 TFs were either greater than 0.1 or smaller than -0.05. On the other hand, cluster 2 for K562 cell-line contained 190 TFs, out of which 108 had effect estimates in the range [-0.01,0.01] and 34 contained effect estimates either greater than 0.01 or smaller than -0.05.


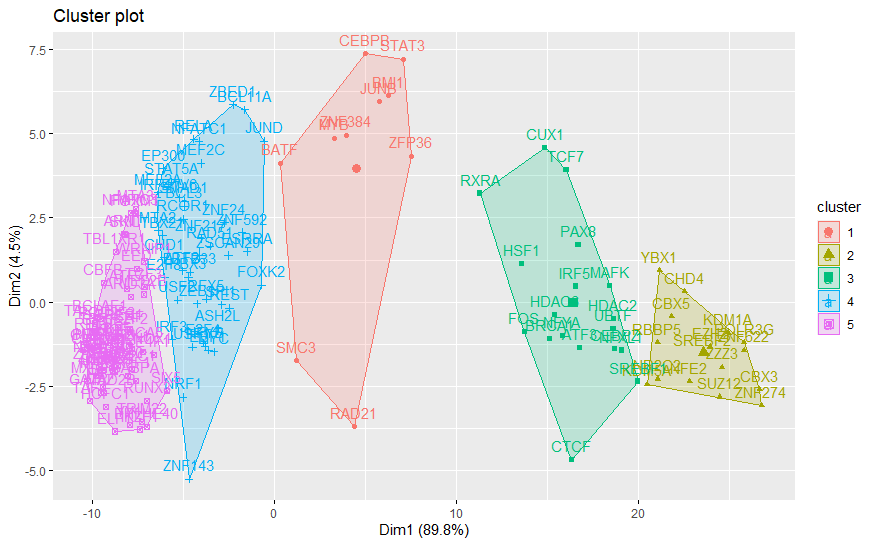

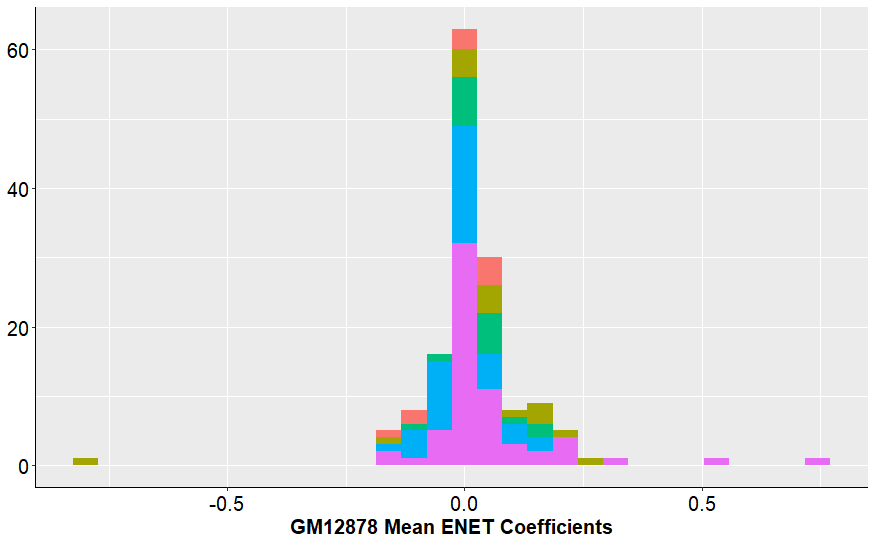

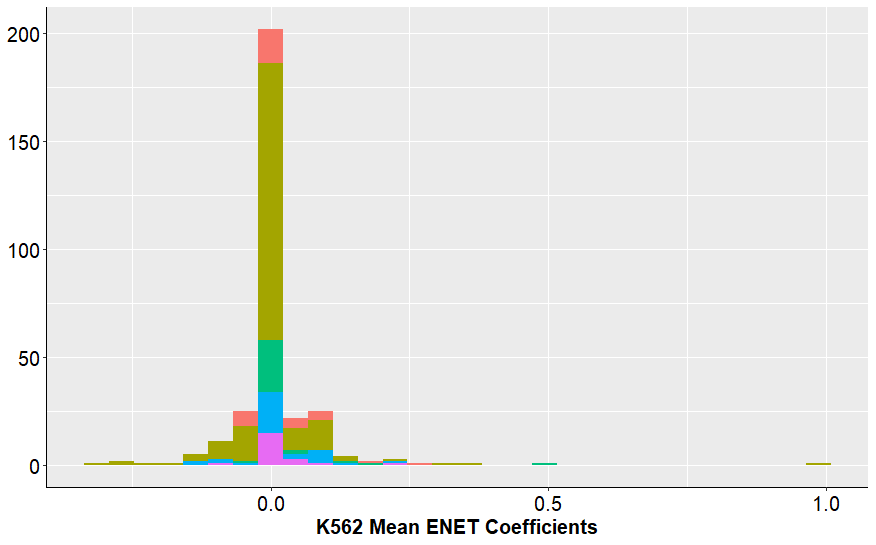

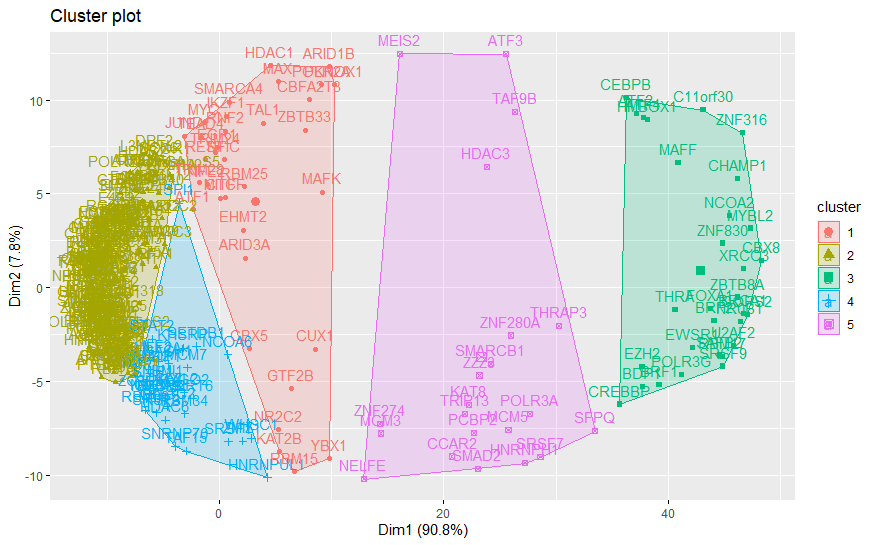


**A**

**B**

**C**

**D**

**Additional Figure 6: The correlation structure for the TFs within PANDA GRN features is captured by ENET models. Plots showing the clusters defined by k-means clustering method using the correlation matrix among the 149 GM12878 TFs in A and the 309 K562 TFs in C. B and D show the histograms containing the mean ENET coefficients for the GM12878 and K562 TFs respectively binned by the clusters in which they were present.**

**References**

[1] A. D. Rouillard *et al.*, “The harmonizome: a collection of processed datasets gathered to serve and mine knowledge about genes and proteins,” *Database*, vol. 2016, Jul. 2016, doi: 10.1093/database/baw100.

[2] H. Han *et al.*, “TRRUST v2: an expanded reference database of human and mouse transcriptional regulatory interactions.,” *Nucleic Acids Res.*, vol. 46, no. D1, pp. D380–D386, Jan. 2018, doi: 10.1093/nar/gkx1013.

[3] H. Zou and T. Hastie, “Regularization and Variable Selection via the Elastic Net,” *J. R. Stat. Soc. Ser. B (Statistical Methodol.*, vol. 67, no. 2, pp. 301–320, Jan. 2005.
