# Supplementary material for: Modeling transcriptional regulation using gene regulatory networks based on multi-omics data sources
Source: BMC Bioinformatics. 2021 Apr 19;22:200. doi: 10.1186/s12859-021-04126-3 (PMC8056605; doi:10.1186/s12859-021-04126-3)
Supplement: Supplementary file 7 — Additional file 7: Supplementary Table S6. Results from fitting the SKAT models. [file 12859_2021_4126_MOESM7_ESM.docx]

**Modeling transcriptional regulation using gene regulatory networks based on multi-omics data sources**

Neel Patel^1,2^ and William S. Bush^2^

^1^Department of Nutrition, Case Western Reserve University, Cleveland, OH, USA. ^2^Department of Population and Quantitative Health Sciences, Case Western Reserve University, Cleveland, OH, USA.

**Supplementary Information**

**Datasets used in the study**

**ChIP-Seq Files**

In order to obtain information about the TFBS across genomes for GM12878 and K562, we used the Encyclopedia of DNA elements(ENCODE) database[1]. We downloaded processed ChIP-seq narrow peak bedfiles corresponding to 153 TFs, 382 and 234 TFs corresponding to GM12878, K562 and HepG2 cell lines respectively that were aligned with hg19/GRCh37 reference assembly of the human genome and that had passed the optimal IDR(Irreproducible Discovery Rate) threshold as defined by the ENCODE consortium. The accession number for these files are provided in table **S1.**

**Gene Annotations**

We used the GRCh37/hg19 reference genome build from the biomaRt library(v.2.44.1)[2] in R to derive gene annotations such as transcription start sites(TSS), length of the gene body, transcript ids, exon ids, transcript lengths, gene ids etc. for all the protein coding genes.

**PWM Files**

We downloaded the position weight matrix(PWM) files corresponding to 469 human TFs from the JASPAR database(v.2020)[3] in MEME format. We later used these files as inputs for running the FIMO algorithm [4]in order to find statistically TFBS across the genome.

**TFBS sequences**

We used the GRCh37/hg19 reference build to obtain sequences corresponding the transcription factor binding sites(TFBS) in the regulatory region of each gene. We later used these sequences as inputs along with the PWMs for running the FIMO algorithm in order to find statistically TFBS across the genome.

**Protein-Protein Interaction Data**

We used the BioGrid database(v.3.5.188)[5] to download PPI data in order to build the PANDA GRNs. We only used the high confidence experimentally validated using experimental techniques such as co-fractionation, co-immunoprecipitation, yeast two-hybrid and affinity capture in BioGrid. We further filtered out the PPIs that did not contain TFs, which ultimately provided us with 1937 PPIs among the GM12878 TFs, 3025 interactions among the K562 TFs and 2807 interactions among HepG2 TFs.

**Co-expression data**

The other source of information that we needed to build the PANDA GRNs was the co-expression matrix. We used different expression data sets in order to build these matrices for the two cell types.

For the GM12878 lymphoblastoid cell line, we used data from the GEUVADIS project[6], which contains lymphoblastoid RNA-seq and genotype data derived from individuals belonging to European and African ancestry groups who participated in the 1000 genomes project. We used the Log normalized expression values(log FPKM) for the 15,785 protein coding genes from the lymphoblastoid cells of 462 individuals in the GEUVADIS dataset with variant effects regressed out using mixed-linear models with a genome-wide genetic relationship matrix(GRM). Our models could be described using the equation below:

  $y=X \beta+Zu+ \epsilon$

Here, y is the vector containing log FPKM expression values for the 462 individuals,  X is the matrix of size 462 by N , where N represents the number of common variants (minor allele frequency > 0.05) present in the dataset (6,326,925), containing the additive genotypes for each variant for each individual,  β is the vector of size N by 1 containing the effect estimates/coefficients of each variant obtained from the fitted regression models;  Z is the GRM of size 462 by 462   built using the number of alleles shared by each pair of individuals at the loci representing all the 6,326,925 variants across the genome; u is the random effects vector of size 462 capturing the random variance for each individual from the GRM and finally  ϵ is the residual vector of size 462 containing the effects not explained by the model. After fitting the models across all the genes, we extracted the ϵ term for each gene which contained the residual expression values. We used these values for building the co-expression matrix.

For the K562 leukemia and the HepG2 hepatocellular carcinoma cell-lines, we downloaded expression data corresponding to four different experiments and five different experiments respectively (accession numbers provided in the supplementary table **S1C**).

K562 expression dataset consisted of 8 different samples while that for HepG2 consisted of 9 different samples. We used the normalized FPKM values corresponding to 12,209 and 13,390 protein coding genes for K562 and HepG2 cell-lines respectively to build the co-expression matrix.

**Processing expression data for ENET prediction models**

We downloaded RNA-seq data for GM12878(ENCSR889TRN), K562(ENCSR545DKY) and HepG2(ENCSR181ZGR) from the Encode database. Each one of these experiments contained processed TG quantification data for two technical replicates. We used the Log10 normalized mean FPKM values as outcome for the ENET prediction models.

**Algorithms used in this study**

**PANDA**

Passing Attributes between Networks for Data Assimilation(PANDA) is a GRN building algorithm developed by Glass et al. to capture information from information corresponding to TF based regulatory mechanisms such as cooperativity among different combinations of TFs and co-regulation of multiple TGs by the same TF in order to weight the regulatory interactions between TF and TGs[7]. It iteratively updates the edge-weights of the regulatory network containing edges between TF and TG by using two Tanimoto similarity based metrics: Responsibility and Availability.

The responsibility of an edge is calculated using the information from the protein-protein interaction(PPI) network, while the availability is calculated from the gene co-expression network. Mathematically, the responsibility of an edge from TF $i$ to its TG $j$ for iteration t, ($R_{\mathrm{ij}}^{(t)}$) is calculated using the following equation:

$$R_{ij}^{(t)}= \frac{\sum_{m} P_{im}^{(t)}W_{mj}^{(t)}}{\sqrt{\sum_{m} \left( P_{im}^{(t)} \right)^{2}+ \sum_{m} \left( W_{mj}^{(t)} \right)^{2}- \left| \sum_{m} P_{im}^{(t)}W_{mj}^{(t)} \right|}}$$

Here, $P_{\mathrm{im}}^{(t)}$is the weight of the edge formed by TF $i$ with another TF $m$ in the PPI network at iteration $t$, while $W_{\mathrm{mj}}^{(t)}$is the edge weight for the connection between $m$and TG $j$in the regulatory network at iteration $t$. The agreement between the PPI and the regulatory network is thus calculated for each edge at each iteration using the cooperativity information among a set of TFs regulating the same set of TGs.

Similarly, the availability of an edge from TF $i$ to its TG $j$ for iteration t, ($A_{\mathrm{ij}}^{(t)}$) is calculated using the co-expression network based on the following equation:

$$A_{ij}^{(t)}= \frac{\sum_{k} W_{ik}^{(t)}C_{kj}^{(t)}}{\sqrt{\sum_{k} \left( W_{ik}^{(t)} \right)^{2}+ \sum_{k} \left( C_{kj}^{(t)} \right)^{2}- \left| \sum_{k} W_{ik}^{(t)}C_{kj}^{(t)} \right|}}$$

Here, $W_{\mathrm{ik}}^{(t)}$ represents the edge weight of the connection between TF $i$ and TG $k$ in the regulatory network and $C_{\mathrm{kj}}^{(t)}$ is the weight of the edge between TGs $k$ and $j$ in the co-expression network. Thus, this equation measures the availability of a TF based on the number of genes that it coregulates.

The weight of the edge between TF $i$ and TG $j$ at each iteration $t$ is then updated by averaging $A_{\mathrm{ij}}^{(t)}$and $R_{\mathrm{ij}}^{(t)}$ as well as a small update parameter $\alpha$:

$$\tilde{W}_{ij}^{(t)}= {0.5R}_{ij}^{(t)}+0.5A_{ij}^{(t)}$$

$$W_{ij}^{(t+1)}=\left( 1-\alpha\right)W_{ij}^{\left( t \right)}+ \alpha\tilde{W}_{ij}^{(t)}$$

Just as information is passed into the regulatory network from PPI and co-expression networks, information is passed out to the two networks using similar methods. The whole process is repeated and the edge-weights are updated until the network reaches convergence, which is determined using Hamming distance:

$$H= \left| \tilde{W}^{(t)}-W^{(t-1)} \right|= \frac{1}{N}\sum_{i,j} \left| \tilde{W}_{ij}^{(t)}- W_{ij}^{(t-1)} \right|$$

Here, $N$ is the number of possible edges in the regulatory network calculated by multiplying the number of TFs and TGs in the network. We used the R package pandaR to implement the PANDA algorithm in our project.

**FIMO**

Find Individual Motif Occurrences(FIMO) is part of the MEME-suite tools for scanning and analysis of TF motifs. It calculates likelihood-ratio score for the occurrence of the motif of individual TF in a given set of DNA sequences, which it then converts into a p-value using dynamic programming using zero order null model of randomly generated background frequencies.[4] It uses bootstrapping to define the false discovery rate(FDR) threshold and identifies significant motif occurrences corresponding to the p-value below that threshold. FIMO requires user to provide the motif information of a TF in the form of one of the variants of position weight matrix(PWM) as well as a set of query DNA sequences to find statistically significant TFBS.

**TEPIC**

TEPIC was developed to predict TFBS based on a biophysical modelling of binding interaction between a TF and DNA motif[8]. It uses transcription affinity prediction(TRAP) algorithm to calculate affinity scores for all the TF motifs within a predefined window around the TSS of a gene. The formula for calculating the TF affinities for each gene is given by:

$$a_{g,i}^{W}= \sum_{p\epsilon P_{W,g}} a_{p,i}e^{-\frac{d_{p,g}}{d_{0}}}s_{p}$$

Here, $a_{g,i}^{W}$ is the total affinity score for TF $i$ binding in the regulatory window $W$ around the TSS of the gene $g$. $P_{W,g}$ is the set of peaks corresponding to TF $i$ in that window, $a_{p,i}$ is the individual affinity scores for each peak $p$ corresponding to $i$ calculated using the TRAP algorithm, $d_{p,g}$ is the distance from the center of peak $p$ to the TSS of $g$ ,it is only used if the window is greater than 50Kb, $d_{0}$ is a constant fixed at 5000Kb and $s_{p}$ is the scaling factor used for peak $p$. TEPIC requires users to provide the annotation files for the genes as well as the bed regions corresponding to TF peaks in order to calculate the TF-gene affinity scores.

**Elastic-Net(ENET) regularized regression**

We used ENET regularized regression models, which linearly combine the lasso(L1) and the ridge(L2) penalty norms for feature selection and handling multi-colinearity among the features, for predicting gene expression.[9]

In the context of gene regulation, ENET uses a combination of two different penalizing methods to find the optimum number of regulators as predictors based on their influence on each other and on the expression of their target gene according the following equation:

|  | $\hat{\beta}=\arg\min_{\beta} \left\Vert y-X\beta\right\Vert+ \alpha\left\Vert\beta\right\Vert^{2}+(1- \alpha)\left\Vert\beta\right\Vert$ | (S1) |
| --- | --- | --- |

Here, $\hat{\beta}$ is the estimated effect coefficient for each input feature(TF), $\beta$ is the effect coefficient for each input feature, $X$ is the input feature matrix containing panda edge weights or tepic, affinity scores, $y$ is the response vector (expression values) and $\alpha$ is the hyperparameter used to control the ratio between the lasso and the ridge penalty norms.

**QBiC-Pred**

We used the Quantitative Predictions of TF Binding Changes Due to Sequence Variants(QBiC-Pred) algorithm to quantify the impact of non-coding variants on TF binding. QBiC-Pred calculates the change in TF binding intensity due to the presence of an alternate allele by using coefficients derived from ordinary least squared(OLS) models trained using 6-mer sequences and *in-vitro* protein binding microarray(uPBM) data. More specifically, difference in the linear combination of the coefficients corresponding to 6-mers present in the 11-bp window around the variant position for the reference and alternate allele is used to compute the effect of the that variant on TF binding(*equation number*).

$$\Delta S= S_{ALT}-S_{REF}$$

$$= \sum_{k=1}^{6} \beta_{i_{k}}-\beta_{j_{k}}$$

Here, $\Delta S$ corresponds to the change in score for TF binding due to the presence of an alternate allele. $\beta_{i_{k}}$ and $\beta_{j_{k}}$are the effect coefficients for k-mer $k$ containing alternate and reference alleles respectively that are derived from the trained OLS models from the *in-vitro* uPBM data.

**SKAT**

Sequence Kernel Association Test(SKAT) is a popular approach for finding association between a group of variants, defined by their membership to a specific functional unit(gene, intron, promoter etc.) and a binary or continuous trait. Given a simple linear regression model for association of a continuous trait $\boldsymbol{y}$ with a group of rare variants $m$ present within a specific region(gene) for $n$ individuals with $p$ covariates:

$$y_{i}= \alpha_{0}+ \boldsymbol{\alpha}^{\boldsymbol{'}}\boldsymbol{X}_{\boldsymbol{i}}+ \boldsymbol{\beta}^{\boldsymbol{'}}\boldsymbol{G}_{\boldsymbol{i}}+ \varepsilon_{i}$$

Here, $y_{i}$ is the continuous outcome trait for *i*-th subject, $\alpha_{0}$ is the intercept term, $\boldsymbol{X}_{\boldsymbol{i}}$ is the vector of covariates $(X_{i1},X_{i2,}X_{i3}\ldots X_{ip})$ and $\boldsymbol{\alpha}^{\boldsymbol{'}}$ is the vector containing each one of their effect coefficients, $\boldsymbol{G}_{\boldsymbol{i}}$ is the vector containing genotypes for $m$ variants $(G_{i1},G_{i2,}G_{i3}\ldots G_{im})$ and $\boldsymbol{\beta}^{\boldsymbol{'}}$ is the effect coefficient vector for each of them. SKAT uses a variance-component score statistic for testing the null hypothesis $H_{0}(\beta_{0}=0)$:

$$Q=\left( \boldsymbol{y}- \hat{\boldsymbol{\mu}} \right)^{'}\boldsymbol{K}(\boldsymbol{y-}\hat{\boldsymbol{\mu}}\boldsymbol{)}$$

Her, $\hat{\boldsymbol{\mu}}$ is the predicted mean of $\boldsymbol{y}$ under $H_{0}$ without using variant information. i.e. $\hat{\boldsymbol{\mu}}= \hat{\alpha}_{0}+ \hat{\boldsymbol{\alpha}}\boldsymbol{X}$**.** SKAT creates a null object to estimate $\hat{\alpha}_{0}$ and $\hat{\boldsymbol{\alpha}}$ by regressing$\boldsymbol{y}$ against the covariate matrix $\boldsymbol{X}$. $\boldsymbol{K}$ is a $n\times n$ similarity matrix, called a kernel matrix, which captures the relatedness between individuals based on the genotypes of $m$ variants. Specifically,

$$\boldsymbol{K}=\boldsymbol{GW}\boldsymbol{G}^{\boldsymbol{'}}$$

$$K\left( G_{i}G_{i}^{'} \right)= \sum_{j=1}^{m} w_{j}G_{ij}G_{i^{'}j}$$

Here, $K(.)$ is the kernel function used to compute the relatedness between individuals, $w_{j}$ is the weight of variant $j$ determined based on some predefined scoring metric.

**Supplementary Methods**

**Creating weighted TFBS motif network**

In order to calculate weighted interaction between TF $t$ and TG $g$ $, W_{\mathrm{tg}}$ in the motif network for the PANDA GRN, we used the following equation:

|  | $W_{tg}=\frac{p_{tg}}{P_{g}}$ | (S2) |
| --- | --- | --- |

Here, $p_{tg}$ is the number of peaks corresponding to $t$ and $P_{g}$ is the total number of peaks for all the TFs present within the cis-regulatory region of TG $g$.

**Prediction performance of the models depended upon the regulatory window defined by using CTCF peaks, but not on the weighting scheme of the TFBS motif network.**

We used the most upstream and downstream CTCF peaks to define the regulatory window of the TGs in order to look for overlapping TFBS. The median regulatory window distance across all the TGs for GM12878, K562 and HepG2 was 46,031 bp, 46,003 bp and 46,099 bp respectively. We also generated a set of TFBS for all the cell-lines based on a 50Kbp window around the gene body to compare the effect of using biologically relevant regulatory windows to those defined using traditional genomic distances on the prediction performance. For all the three cell-lines, the 50Kbp based TFBS set were significantly larger compared to the ones based on CTCF peaks (GM12878: 1,170,644 additional TFBS; K562: 2,096,025 additional TFBS and HepG2: 1,607,755 additional TFBS). In spite of this difference, the prediction performance of the CTCF defined TFBS based GRNs was significantly better to that obtained from the GRNs constructed using a traditional 50Kbp window to find TFBS as shown in **Supplementary Figure S1**. Thus, using biologically defined regulatory boundaries for finding TFBS is essential to build GRNs and predict gene expression.

**Supplementary Figure S1: Influence of using CTCF defined regulatory windows on gene expression prediction. Boxplots showing results from predicting gene expression using GRNs built using TFBS defined based on gene regulatory windows based upon CTCF peaks vs. 50Kb regions around TG body with respect to A)PCC and B)MSE**


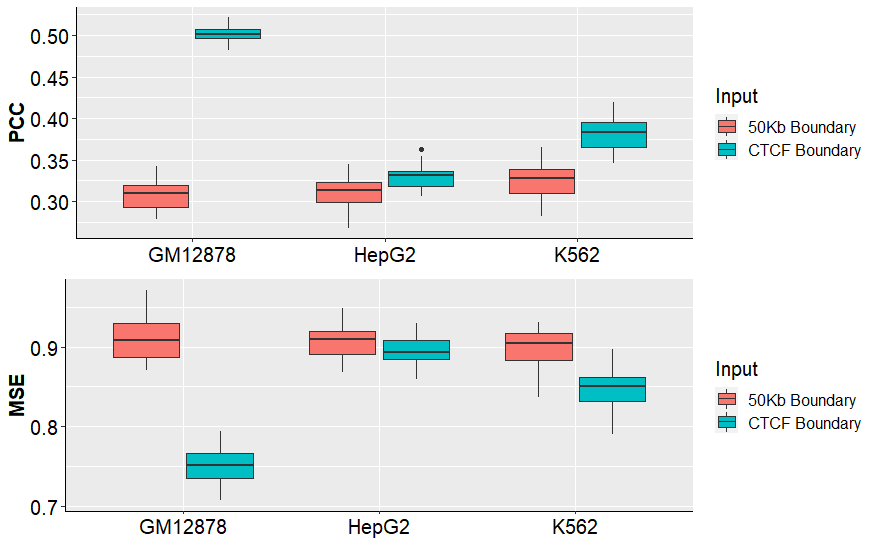


***

***

ns

***

***

**

**A**

**B**

**Figure S4: GO Enrichment results for the TFs placed in different bins for the two cell types: We divided the TFs into 5 bins based on their average effect estimates. A) shows the top 5 significant GO BP and GO MF enrichment terms for 149 GM12878 TFs and B) shows the same for 309 K562 TFs. The complete set of significant enrichment terms have been provided in supplementary table number.**


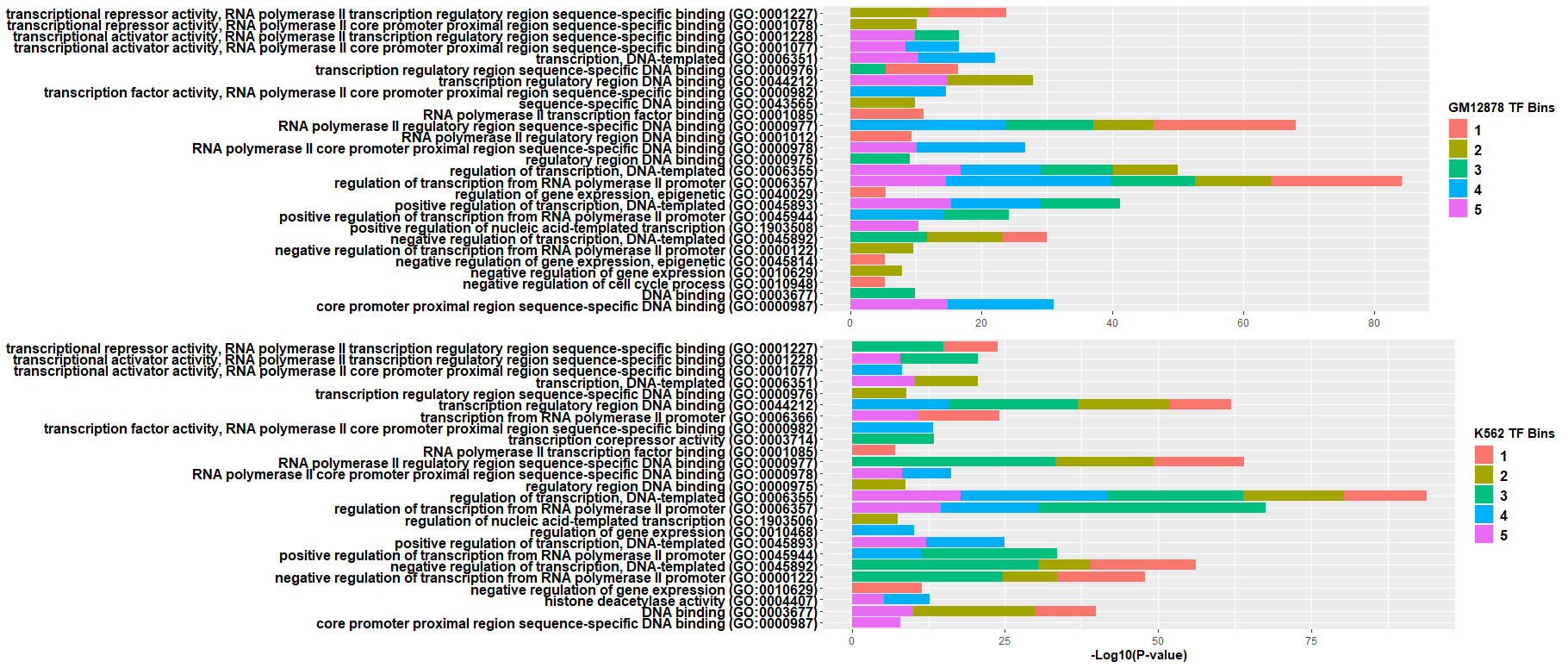


**Supplementary Figure S3: GO Enrichment results for the TFs placed in different bins for the two cell types: We divided the TFs into 5 bins based on their average effect estimates. A) shows the top 5 significant GO BP and GO MF enrichment terms for 149 GM12878 TFs and B) shows the same for 309 K562 TFs. The complete set of significant enrichment terms have been provided in supplementary tables S4C and S4D**

**A**

**B**


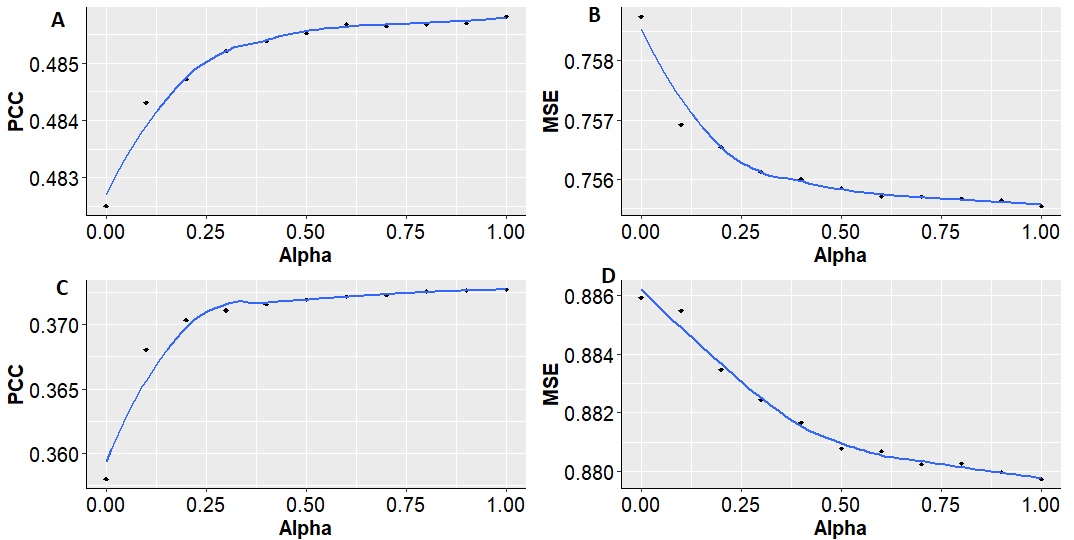


**Supplementary Figure S2: A) and B) show the prediction performance of the ENET regression models with regards to PCC and MSE respectively for different values of alpha(“l1_ratio”) for 1 iteration built after predicting expression for GM12878 TGs . C) and D) show the performance for PCC and MSE respectively after predicting expression for K562 TGs.**

**Comparison of the TF effect estimates learned from the TEPIC GRN and TEPIC models**

We extracted the effect estimates for 80 TFs(GM12878) and 86 TFs(K562) learned from the ENET prediction tasks based on TEPIC GRN edge-weights and TEPIC scorers over 20 iterations. We averaged these estimates based on equation (1) and created a rank ordered list of TFs indicating their influence over the predictive performance of the models. We then compared the ranks from the TEPIC score based models to those obtained from TEPIC GRN models. 73 of the 80 GM12878 TFs and 76 of the 86 K562 TFs had their ranks changed between the two models. Of these we observed a change of rank of at least 10 positions in either direction for 33 GM12878 TFs and 38 K562 TFs as described in **Supplementary Table S2.** The data corresponding to all the TFs is described in **Supplementary Tables S5A** and **S5B.** We observed that TFs for both cell-lines that were placed significantly higher based on TEPIC GRN models compared to the TEPIC models, indicated by positive change in ranks, are involved in transcriptional activation of many protein coding genes. These TFs are MAX, NR2C2, POU2F2,

RELA, RELB and ETS1 for GM12878 and CEBP1, SPI1, PKNOX1, MYBL2 and NFE2 for K562. On the other hand, TFs with a significant negative change in ranks between the two models consisted of transcriptional repressors such as BHLHE40, RXRA and SREBF1 for GM12878 and MITF, NFIC and THAP1 for K562. We also note that we did not observe any change in ranks for most of the top and bottom ranked TFs for both cell-types. Thus, using TEPIC scores alone provided important information for building prediction models which was further enhanced by using GRN weightings on top of these scores.

**Supplementary Table S2: The mean ENET effect estimates, ranks and the change in ranks for the 33 TFs(GM12878) and 38 TFs(K562) obtained from comparing the TEPIC and TEPIC GRN models. Here, TFs with the change in rank of at least 10 positions are shown.**


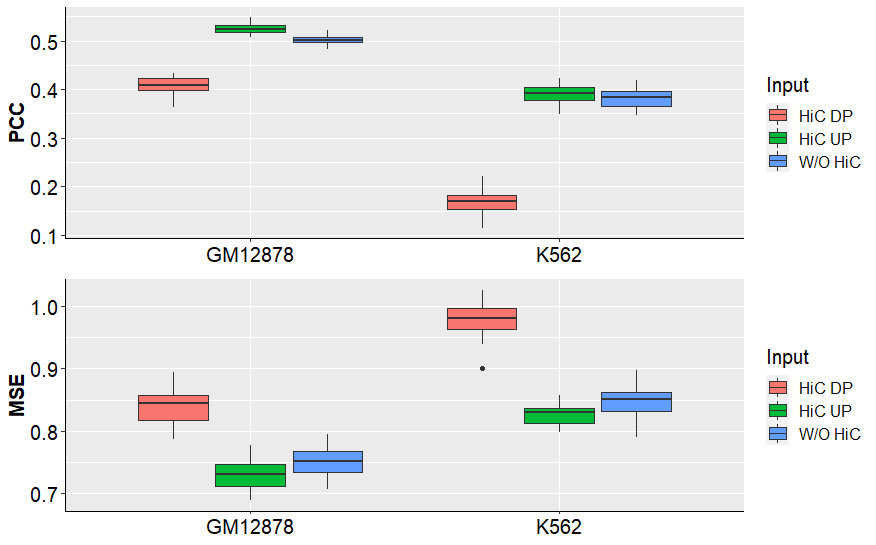


**A**

**B**

**Supplementary Figure S4: Capturing the effect of long distance interactions between TF peaks and TG promoters on expression prediction. This figure is the extension of Figure-5C with the results from HiC DP GRN based prediction models added to the analysis. The prediction performance of HiC UP GRN prediction models was significantly better than the ones constructed without HiC information and the HiC DP normalized TF-TG motif network. A shows the prediction performance w.r.t median PCC while B shows the performance w.r.t median MSE. differently weighted HiC motif matrices ( HiC DP and HiC UP) in comparison to those using GRNs based on unweighted motif matrices(Pos GRNs) for both cell types.**


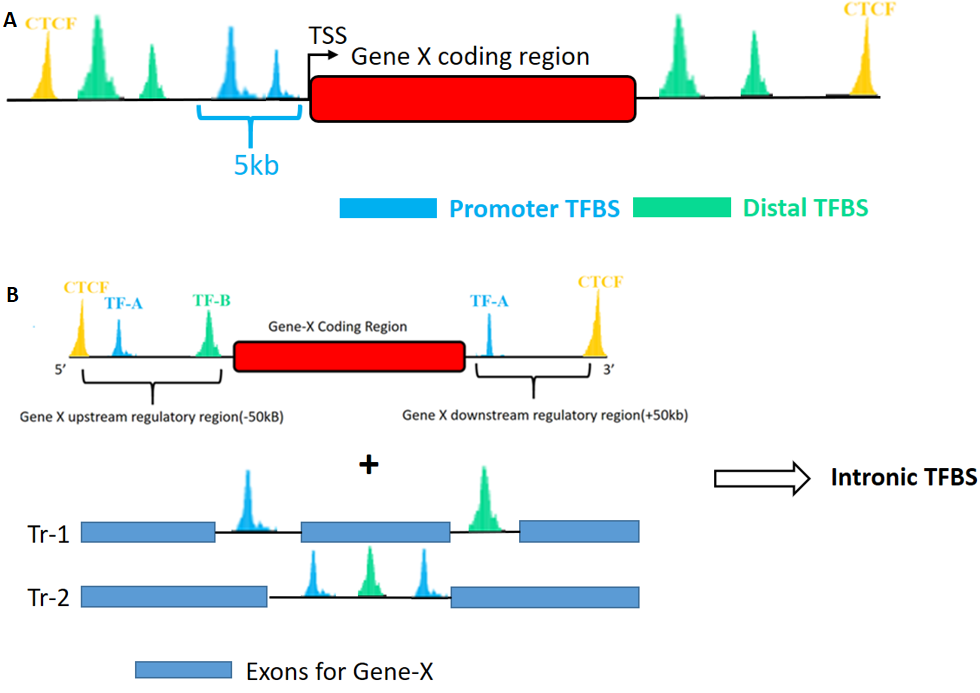


**Supplementary Figure S5: Identifying TFBS in different regulatory elements. A) We used the 5Kb region upstream of the TSS for each gene to identify promoter TFBS. B) We extracted all the TFBS outside of the promoter region to isolate the distal TFBS. The number of promoter and distal TFBS for both cell-types have been provided in Table-1.**

| ***Descriptions of the supplementary tables attached with the manuscript***  **S1A** | **ENCODE accessions for the GM12878 ChIP-Seq files** |
| --- | --- |
| **S1B** | **ENCODE accessions for the K562 ChIP-Seq files** |
| **S1C** | **ENCODE accessions for the HepG2 ChIP-Seq files** |
| **S1D** | **ENCODE accessions for the K562 and HepG2 RNA-seq experiments used for PANDA co-expression network** |
| **S3A** | **Median PCC and MSE calculated from GM12878 based prediction models.** |
| **S3B** | **Median PCC and MSE calculated from K562 based prediction models** |
| **S3C** | **Median PCC and MSE calculated from HepG2 based prediction models** |
| **S3D** | **Wilcoxon rank sum test p-values calculated using the median MSE comparisons among GM12878 prediction models.** |
| **S3E** | **Wilcoxon rank sum test p-values calculated using the median PCC comparisons among GM12878 prediction models.** |
| **S3F** | **Wilcoxon rank sum test p-values calculated using the median MSE comparisons among K562 prediction models.** |
| **S3G** | **Wilcoxon rank sum test p-values calculated using the median PCC comparisons among K562 prediction models.** |
| **S3H** | **Wilcoxon rank sum test p-values calculated using the median MSE comparisons among HepG2 prediction models.** |
| **S3I** | **Wilcoxon rank sum test p-values calculated using the median PCC comparisons among HepG2 prediction models.** |
| **S4A** | **Mean ENET effect estimates calculated from 20 iterations of the GM12878 Pos GRN based prediction models along with their respective bins** |
| **S4B** | **Mean ENET effect estimates calculated from 20 iterations of the K562 Pos GRN based prediction models along with their respective bins** |
| **S4C** | **GO enrichment results for biological processes and molecular functions for GM12878 TFs in each bin created using their average effect estimates.** |
| **S4D** | **GO enrichment results for biological processes and molecular functions for K562 TFs in each bin created using their average effect estimates.** |
| **S5A** | **GM12878 TF ranks based on mean effect estimates calculated using TEPIC and TEPIC GRN prediction models** |
| **S5B** | **K562 TF ranks based on mean effect estimates calculated using TEPIC and TEPIC GRN prediction models** |
| **S6A** | **SKAT results for DGN QBiC-Pred z-score models** |
| **S6B** | **SKAT results for DGN GRN merge score models** |
| **S6C** | **SKAT results for GTEx replication analysis** |

**References**

[1] C. A. Davis *et al.*, “The Encyclopedia of DNA elements (ENCODE): data portal update,” *Nucleic Acids Res.*, vol. 46, no. D1, pp. D794–D801, Nov. 2017, doi: 10.1093/nar/gkx1081.

[2] S. Durinck *et al.*, “BioMart and Bioconductor: a powerful link between biological databases and microarray data analysis.,” *Bioinformatics*, vol. 21, no. 16, pp. 3439–3440, Aug. 2005, doi: 10.1093/bioinformatics/bti525.

[3] O. Fornes *et al.*, “JASPAR 2020: update of the open-access database of transcription factor binding profiles,” *Nucleic Acids Res.*, vol. 48, no. D1, pp. D87–D92, Nov. 2019, doi: 10.1093/nar/gkz1001.

[4] C. E. Grant, T. L. Bailey, and W. S. Noble, “FIMO: scanning for occurrences of a given motif,” *Bioinformatics*, vol. 27, no. 7, pp. 1017–1018, Feb. 2011, doi: 10.1093/bioinformatics/btr064.

[5] R. Oughtred *et al.*, “The BioGRID interaction database: 2019 update,” *Nucleic Acids Res.*, vol. 47, no. D1, pp. D529–D541, Nov. 2018, doi: 10.1093/nar/gky1079.

[6] T. Lappalainen *et al.*, “Transcriptome and genome sequencing uncovers functional variation in humans,” *Nature*, vol. 501, no. 7468, pp. 506–511, 2013, doi: 10.1038/nature12531.

[7] K. Glass, C. Huttenhower, J. Quackenbush, and G.-C. Yuan, “Passing Messages between Biological Networks to Refine Predicted Interactions,” *PLoS One*, vol. 8, no. 5, p. e64832, May 2013.

[8] F. Schmidt *et al.*, “Combining transcription factor binding affinities with open-chromatin data for accurate gene expression prediction,” *Nucleic Acids Res.*, vol. 45, no. 1, pp. 54–66, Nov. 2016, doi: 10.1093/nar/gkw1061.

[9] H. Zou and T. Hastie, “Regularization and Variable Selection via the Elastic Net,” *J. R. Stat. Soc. Ser. B (Statistical Methodol.*, vol. 67, no. 2, pp. 301–320, Jan. 2005.

**Additional Acknowledgements:**

**Depression Genes Network Data**

**Study 7 (GenRED I)**

**Data and biomaterials were collected in six projects that participated in the National Institute of Mental Health (NIMH) Genetics of Recurrent Early-Onset Depression (GenRED) project. From 1999-2003, the Principal Investigators and Co-Investigators were: New York State Psychiatric Institute, New York, NY, R01 MH060912, Myrna M. Weissman, Ph.D. and James K. Knowles, M.D., Ph.D.; University of Pittsburgh, Pittsburgh, PA, R01 MH060866, George S. Zubenko, M.D., Ph.D. and Wendy N. Zubenko, Ed.D., R.N., C.S.; Johns Hopkins University, Baltimore, MD, R01 MH059552, J. Raymond DePaulo, M.D., Melvin G. McInnis, M.D. and Dean MacKinnon, M.D.; University of Pennsylvania, Philadelphia, PA, RO1 MH61686, Douglas F. Levinson, M.D. (GenRED coordinator), Madeleine M. Gladis, Ph.D., Kathleen Murphy-Eberenz, Ph.D. and Peter Holmans, Ph.D. (University of Wales College of Medicine); University of Iowa, Iowa City, IW, R01 MH059542, Raymond R. Crowe, M.D. and William H. Coryell, M.D.; Rush University Medical Center, Chicago, IL, R01 MH059541-05, William A. Scheftner, M.D., Rush-Presbyterian.**

**Study 18**

**Data and biomaterials were obtained from the limited access datasets distributed from the NIH-supported "Sequenced Treatment Alternatives to Relieve Depression" (STAR*D). STAR*D focused on non-psychotic major depressive disorder in adults seen in outpatient settings. The primary purpose of this research study was to determine which treatments work best if the first treatment with medication does not produce an acceptable response. The study was supported by NIMH Contract # N01MH90003 to the University of Texas Southwestern Medical Center. The ClinicalTrials.gov identifier is NCT00021528.**

**Study 20 (TADS and SOFTAD)**

**TADS (Treatment for Adolescents with Depression Study) was supported by Contract N01 MH80009 from the National Institute of Mental Health to Duke University Medical Center (John S. March, Principle Investigator). The authors would like to thank the members of the TADS Team. TADS is coordinated by the Department of Psychiatry and Behavioral Sciences and the Duke Clinical Research Institute at Duke University Medical Center in collaboration with the National Institute of Mental Health (NIMH), Rockville, Maryland. The Coordinating Center principal investigators are John March, Susan Silva, Stephen Petrycki, John Curry, et al.**

**SOFTAD (Substance Use and Othe Outcomes Following Treatment for Adolecent Depression) was funded by grant R01 MH070494 from the National Institute of Mental Health to John Curry, Ph.D. We are indebted to Benedetto Vitiello, M.D. who coordinated administration of SOFTAD at NIMH. We thank participants and the site staff who recruited them, including Margaret Price, Stephanie Frank, and Sue Baab. We acknowledge the many contributions of the late Dr. Elizabeth Weller, a dedicated clinical scientist.**

**Study 52 (GenRED II)**

**Data and biomaterials in this release were collected in six projects that participated in the National Institute of Mental Health (NIMH) Genetics of Recurrent Early-Onset Depression (GenRED) project (1999-2009). The Principal Investigators and Co-Investigators were: New York State Psychiatric Institute, New York, NY, R01 MH 060912, Myrna M. Weissman, Ph.D.; Johns Hopkins University, Baltimore, MD, R01 MH059552, J. Raymond DePaulo, M.D., and James B. Potash, M.D., M.P.H.; University of Pennsylvania, Philadelphia, PA (1999-2005), and Stanford University (2006-2009), R01 MH61686, Douglas F. Levinson, M.D. (GenRED coordinator); University of Iowa, Iowa City, IW, R01 MH059542e, Raymond R. Crowe, M.D., and William H. Coryell, M.D.; Rush University Medical Center, Chicago, IL, R01 MH059541-05, William A. Scheftner, M.D.; and University of Pittsburgh, Pittsburgh, PA (1999-2003), R01 MH060866, George S. Zubenko, M.D., Ph.D., and Wendy N. Zubenko, Ed.D., R.N., C.S.**

**Study 73 (CO-MED)**

**Data used in the preparation of this article were obtained from the limited access datasets distributed from the NIH-supported "Combing Medications to Enhance Depression Outcomes" (CO-MED). This is a multisite, clinical trial of persons with depression comparing the effectiveness of randomly assigned medication treatment. The study was supported by NIMH Contract # N01 MH090003-02 to the University of Texas Soutwestern Medical Center. The ClinicalTrials.gov identifier is NCT00590863.**

**Study 83**

**Data and biomaterials collected for project "Incomplete Response in Late Life Depression: Getting to Remission (IRL GREY)". This project was supported by ClinicalTrials.gov Identifier: NCT00892047 and 5R01MH083660-05 from the National Institute of Mental Health (NIMH). Principal Investigators are: Charles F. Reynolds, M.D., University of Pittsburgh (Responsible PI); Eric Lenze, M.D., Washington University School of Medicine, St. Louis; and Benoit Mulsant, M.D., University of Toronto.**

**Study 84**

**NIMH Study 84 (Site 276) was funded by two independent NIMH grants. The principal investigators for the Emory CIDAR center grant were supported by funding from the National Institute for Mental Health grant P50MH077083 (Helen Mayberg). Additional funding was obtained for long-term follow-up of study participants via R01MH080880 (W. Edward Craighead). Additional Emory investigators who contributed to this study were Boadie Dunlop, Elisabeth Binder (Emory/Max Planck Institute Munich), Joseph Cubells, Xiaoping Hu, Mary Kelley, Clint Kilts (now University of Arkansas for Medical Sciences), Becky Kinkead, Michael Owens, Drew Westen, Thaddeus Pace (now University of Arizona), Charles B. Nemeroff (now University of Miami), and James Ritchie.**

**Study 88**

**Data was provided by Dr. Douglas F. Levinson. We gratefully acknowledge the resources were supported by National Institutes of Health/National Institute of Mental Health grants 5RC2MH089916 (PI: Douglas F. Levinson, M.D.; Co-investigators: Myrna M. Weissman, Ph.D., James B. Potash, M.D., MPH, Daphne Koller, Ph.D., and Alexander E. Urban, Ph.D.) and 3R01MH090941 (Co-investigator: Daphne Koller, Ph.D.).**

**Study 108**

**Data and biomaterials were collected as part of the National Institutes of Health-funded study 'Sustaining Remission of Psychotic Depression' (5U01MH062446, 5U01MH062518, and 5U01MH062624). Study sites were Weill Medical College of Cornell University and New York Presbyterian Hospital, Westchester Division, NY (PIs: Drs. Barnett Meyers and George Alexopoulos); the University of Massachusetts Medical School and UMass Memorial Health Care, Worcester, MA (PI: Dr. Anthony Rothschild); Western Psychiatric Institute and Clinic, Department of Psychiatry, University of Pittsburgh School of Medicine, Pittsburgh, PA (PI: Dr. Ellen Whyte); and the Departments of Psychiatry, University of Toronto and University Health Network, Toronto, Canada (PI: Dr. Alastair Flint). The investigators are very grateful to the patients who participated in and contributed to the study.**

**Genotype Tissue Expression Project Data**

**The Genotype-Tissue Expression (GTEx) Project was supported by the Common Fund of the Office of the Director of the National Institutes of Health (commonfund.nih.gov/GTEx). Additional funds were provided by the NCI, NHGRI,**

**NHLBI, NIDA, NIMH, and NINDS. Donors were enrolled at Biospecimen Source Sites funded by NCI Leidos Biomedical Research, Inc. subcontracts to the National Disease Research Interchange (10XS170), Roswell Park Cancer Institute (10XS171), and Science Care, Inc. (X10S172). The Laboratory, Data Analysis, and Coordinating Center (LDACC) was funded through a contract (HHSN268201000029C) to the The Broad Institute, Inc. Biorepository operations were funded through a Leidos Biomedical Research, Inc. subcontract to Van Andel Research Institute (10ST1035). Additional data**

**repository and project management were provided by Leidos Biomedical Research, Inc.(HHSN261200800001E). The Brain Bank was supported supplements to University of Miami grant DA006227. Statistical Methods development grants were made to the University of Geneva (MH090941 & MH101814), the University of Chicago (MH090951,MH090937, MH101825, & MH101820), the University of North Carolina - Chapel Hill (MH090936), North Carolina State University (MH101819), Harvard University (MH090948), Stanford University (MH101782), Washington University (MH101810), and to the University of Pennsylvania (MH101822). The datasets used for the analyses described in this manuscript were obtained from dbGaP at http://www.ncbi.nlm.nih.gov/gap through dbGaP accession number phs000424.v3.p1.**

**References**
